# Haploinsufficiency of *Dmxl2*, Encoding a Synaptic Protein, Causes Infertility Associated with a Loss of GnRH Neurons in Mouse

**DOI:** 10.1371/journal.pbio.1001952

**Published:** 2014-09-23

**Authors:** Brooke Tata, Lukas Huijbregts, Sandrine Jacquier, Zsolt Csaba, Emmanuelle Genin, Vincent Meyer, Sofia Leka, Joelle Dupont, Perrine Charles, Didier Chevenne, Jean-Claude Carel, Juliane Léger, Nicolas de Roux

**Affiliations:** 1 Inserm, U1141, Paris, France; 2 Université Paris Diderot, Sorbonne Paris Cité, Hôpital Robert Debré, Paris, France; 3 Inserm, U1078, Brest, France; 4 Genoscope, IG-DSV-CEA, Evry, France; 5 INRA, UMR85 Physiologie de la Reproduction et des Comportements, Nouzilly, France; 6 Genetics Department and Inserm US975, Université Pierre et Marie Curie, Hôpital la Pitié-Salpêtrière, Paris, France; 7 AP-HP, Laboratoire de Biochimie, Hôpital Robert Debré, Paris, France; 8 AP-HP, Service d'Endocrinologie Diabétologie Pédiatrique et Centre de Référence des Maladies Endocriniennes Rares de la Croissance, Hôpital Robert Debré, Paris, France; University of Cambridge, United Kingdom

## Abstract

Characterization of the genetic defects causing gonadotropic deficiency has made a major contribution to elucidation of the fundamental role of Kisspeptins and Neurokinin B in puberty onset and reproduction. The absence of puberty may also reveal neurodevelopmental disorders caused by molecular defects in various cellular pathways. Investigations of these neurodevelopmental disorders may provide information about the neuronal processes controlling puberty onset and reproductive capacity. We describe here a new syndrome observed in three brothers, which involves gonadotropic axis deficiency, central hypothyroidism, peripheral demyelinating sensorimotor polyneuropathy, mental retardation, and profound hypoglycemia, progressing to nonautoimmune insulin-dependent diabetes mellitus. High-throughput sequencing revealed a homozygous in-frame deletion of 15 nucleotides in *DMXL2* in all three affected patients. This homozygous deletion was associated with lower *DMXL2* mRNA levels in the blood lymphocytes of the patients. *DMXL2* encodes the synaptic protein rabconnectin-3α, which has been identified as a putative scaffold protein for Rab3-GAP and Rab3-GEP, two regulators of the GTPase Rab3a. We found that rabconnectin-3α was expressed in exocytosis vesicles in gonadotropin-releasing hormone (GnRH) axonal extremities in the median eminence of the hypothalamus. It was also specifically expressed in cells expressing luteinizing hormone (LH) and follicle-stimulating hormone (FSH) within the pituitary. The conditional heterozygous deletion of *Dmxl2* from mouse neurons delayed puberty and resulted in very low fertility. This reproductive phenotype was associated with a lower number of GnRH neurons in the hypothalamus of adult mice. Finally, *Dmxl2* knockdown in an insulin-secreting cell line showed that rabconnectin-3α controlled the constitutive and glucose-induced secretion of insulin. In conclusion, this study shows that low levels of DMXL2 expression cause a complex neurological phenotype, with abnormal glucose metabolism and gonadotropic axis deficiency due to a loss of GnRH neurons. Our findings identify rabconectin-3α as a key controller of neuronal and endocrine homeostatic processes.

## Abstract 2

Rabconnectin-3α and the control of puberty Human genetics shows that low levels of rabconnectin-3α cause a loss of the neurons that produce gonadotropin-releasing hormone, revealing a new mechanism for incomplete puberty and infertility.

## Author Summary

Investigation of neurodevelopmental disorders with abnormal puberty can reveal neuronal processes that control the initiation of puberty and subsequent reproductive function. We describe here a new syndrome observed in three brothers with incomplete puberty, central hypothyroidism, peripheral polyneuropathy, mental retardation, and abnormal glucose regulation. Molecular genetic investigation in this consanguineous family revealed an in-frame deletion of 15 nucleotides in the *DMXL2* gene, which resulted in lower levels of its expression. We found that rabconnectin-3α, the synaptic protein encoded by *DMXL2*, is widely expressed in the brain and in the ends of the axons of neurons that produce gonadotropin-releasing hormone (GnRH). We then observed that neuron-specific deletion of one allele of *Dmxl2* in mice reproduces the incomplete puberty seen in the human patients and results in decreased numbers of GnRH neurons in the hypothalamus. We also showed that rabconnectin-3α controls glucose-induced insulin secretion. These findings reveal a new mechanism of gonadotropin deficiency and identify rabconnectin-3α as a key controller of neuronal and endocrine homeostasis.

## Introduction

Puberty is defined by the appearance of secondary sexual characteristics and the maturation of reproductive function. It is driven by an increase in sexual steroid hormone synthesis by the gonads, under the control of the hypothalamo–pituitary hormonal axis [1]. The key event in puberty initiation is an increase in the pulsatile release of GnRH by hypothalamic neurons, triggering the release of luteinizing hormone (LH) and follicle-stimulating hormone (FSH) by the pituitary gland [2]. This pubertal increase in GnRH secretion is associated with increases in glutamatergic inputs and decreases in GABAergic inputs to GnRH neurons [3]. It is also facilitated by hypothalamic glial cells, which interact directly or indirectly with GnRH neurons [4]. Kisspeptins (KPs) are currently thought to be the principal hypothalamic neuropeptides controlling GnRH secretion in mammals, not only at puberty but also during adulthood [5],[6]. The activation of the KP signaling pathway in the hypothalamus is seen as the major hallmark of puberty onset [5],[6], and its control is partly epigenetic [7].

Congenital hypogonadotropic hypogonadism (CHH) is a medical condition defined by a defect of the hypothalamo–pituitary–gonadotropic (HPG) axis resulting in an absence of puberty and infertility in adulthood. Characterization of the genetic defects underlying the isolated form of CHH [8]–[12] has proved crucial, not only for elucidating the fundamental role of KPs in the central regulation of the gonadotropic axis and puberty [5],[6] but also for determining the role played by Neurokinin B (NKB) [13]. CHH may be associated with anosmia, due to olfactory bulb agenesis, in Kallmann syndrome (KS) [14],[15]. In KS, GnRH neurons fail to leave the olfactory placode (OP). Indeed, GnRH neurons, unlike other neurons of the CNS, migrate early in embryogenesis, from the OP into the hypothalamus, along the olfactory tract [16]. All genes linked to KS encode proteins necessary for olfactory bulb development or for the migration and maturation of GnRH neurons [15].

CHH has also been described in highly complex neurodevelopmental disorders caused by loss-of-function mutations in genes encoding proteins involved in diverse cellular pathways [17]–[21]. This association indicates that the same etiopathogenic mechanism may be responsible for a specific neuroendocrine deficiency and common neurological dysfunctions. We recently decided to investigate these common mechanisms further, by characterizing the molecular mechanism of a new syndrome that associates GnRH deficiency with complex neurological and endocrine phenotypes. A genetic analysis of this family revealed that this phenotype was due to a weak expression of *DMXL2*, encoding Rabconnectin-3α. Through in vivo and in vitro analysis of rabconnectin-3α expression and function, we discovered that weak *Dmxl2* expression in the adult mouse brain resulted in a loss of GnRH neurons in the hypothalamus. These findings established a novel molecular mechanism leading to gonadotropic axis deficiency and paved the way for improvements in our understanding of the neuroendocrine control of reproduction.

## Results

### Phenotype

**Figure 1:**
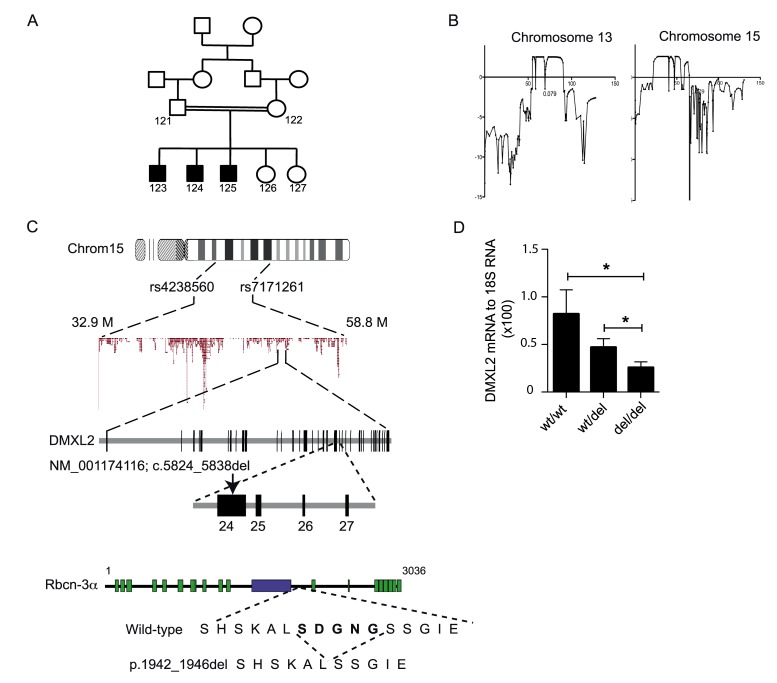
*DMXL2* is mutated in affected patients. (A) Pedigree of the affected family. Closed symbols indicate affected individuals. (B) Linkage analysis delineated two candidate regions on chromosomes 13 and 15 with a LOD score of 2.5. (C) Next-generation sequencing characterized a deletion of 15 nucleotides (c.5824_5838del) in exon 24 of *DMXL2*. This deletion removes five amino acids (p.1942_1946del). Rbcn-3α is a protein with 17 WD domains (green box) and one Rav1p_C domain, which is involved in regulating the glucose-dependent assembly and disassembly of the V1 and V0 subunits of the vacuolar ATPase (purple box) [57]. (D) Quantification, by RT-qPCR, of *DMXL2* mRNA levels relative to RNA 18S levels in blood lymphocytes. Error bars, SEM. * *p*<0.05. Numerical data used to generate graph 1D may be found in Table S5.

We investigated a consanguineous Senegalese family with five children, three of whom had an unusual, progressive endocrine and neurodevelopmental disorder, with similar clinical features in each case (Figure 1A and Table 1). This syndrome began early in childhood, with growth retardation and recurrent episodes of profound asymptomatic hypoglycemia (blood glucose concentration as low as 1.6 mmol/l), with an incomplete suppression of insulin concentrations (2 to 4 mIU/l), appearing between the ages of 2 and 5 ys. Between the ages of 14 and 16 y, the three brothers gradually developed progressive nonautoimmune insulin-dependent diabetes mellitus. Their puberty was incomplete and they had a low testicular volume. All the brothers had a normal sense of smell. During adolescence, the brothers had walking difficulties, such as ataxia and dysarthria. Neurological examination showed polyneuropathy, with a motor deficit predominantly affecting the proximal lower limbs, pes cavus, and claw toes, together with cerebellar and pyramidal signs. Electrophysiological patterns were consistent with a demyelinating polyneuropathy, with a diffuse homogeneous pattern of slowing motor conduction in peripheral nerves (23–42 m/s; N>50 m/s) and greater preservation of sensory conduction in the legs than in the arms. Brain MRI showed moderate subcortical temporal white matter disease, and one patient (no. 123) also had mild hypoplasia of the cerebellum and a small anterior pituitary. The posterior lobe of the pituitary was in the correct position.

**Table 1:** Summary of the clinical phenotype of the three affected patients with *DMXL2* mutations (see Figure 1 for patient numbering).

*Patient*	*#123*	*#124*	*#125*
Age (years)	21.4	20.3	16.8
Weight at birth g (SDS)	2,700 (−2.2)	2,810 (−0.9)	3,060 (−1.1)
Height at birth cm (SDS)	46 (−2.5)	47 (−1.1)	49 (−0.8)
Postnatal growth retardation	+	+	+
Height cm (SDS)	159 (−2.8)	165 (−1.8)	156 (−2.5)
Body mass index kg/m^2^ (SDS)	17.4 (−1.9)	21.3 (−0.2)	15.2 (−2.0)
Head circumference, cm (SDS)	54.5 (−1.9)	56 (−0.8)	51.5 (−2.5)
Ataxia	+	+	+
Dystonia	+	+	+
Demyelinating sensorimotor polyneuropathy	+	+	+
Pyramidal syndrome	+	+	+
Motor delay	+	+	+
Dysarthria	+	+	+
Partial frontal alopecia	+	+	+
Intellectual disability (moderate)	+	+	+
White matter disease (moderate subcortical, temporal)	+	+	+
Hypoglycemia during childhood (asymptomatic)	+	+	+
Nonautoimmune insulin-dependent diabetes mellitus	+	+	+
Hypogonadotropic hypogonadism	+	+	+
Low testicular volume (normal values in young adults >12 ml)	5 ml	7 ml	8 ml
Central hypothyroidism	+	+	+
Hypoplastic pituitary gland	+	−	−
Other features		Progressive hearing loss	

SDS, standard deviation score.

Hormonal assays in adulthood showed an increase in HbA1c levels to up to 11.7%, with low insulin concentrations in an intravenous glucose tolerance test and subnormal basal C-peptide secretion; serum-free T4 concentrations were low, with normal TSH concentrations (Table 2). The effects of TRH administration indicated that this central hypothyroidism was probably of hypothalamic origin. All three brothers had low plasma testosterone concentrations for their age, along with low LH and FSH concentrations. A GnRH test (100 µg i.v.) was performed in two patients (nos. 123 and 124 at 21 and 20 y of age, respectively). It revealed a large increase in LH concentrations associated with a normal increase in FSH concentrations (Table 2).

**Table 2:** Hormonal status of the three affected patients.

*Patient*	*#123*	*#124*	*#125*
Age (years)	21.4	20	16.8
Plasma testosterone concentration (ng/ml)	1.6	1.9	3.0
FSH (IU/l) basal–peak (after GnRH administration)	6.2–16.2	8.3–28	—
LH (IU/l) basal–peak (after GnRH administration)	6.2–57	4.5–71	—
Inhibin B (pg/ml)	65	87	163
FT4 (pmol/l)	8.5	8.3	8.8
TSH (mIU/l) basal and TRH test	1.7–21.9–7.2	1.5–14–5.6	5.5–29–12.4
Prolactin (ng/ml)	13	12	—
HbA1c %	11	11.7	7.1
Insulinemia (mU/l) (IGTT, T1+T3 min)	11	29	9
C-peptide (nmol/l), fasting concentration	0.31	0.93	0.33

Testosterone, 3–12 ng/ml; GnRH test (100 µg i.v.): normal values for FSH, 1–8.5 IU/l; LH, 1.5–8 IU/l. 60 minutes after GnRH administration, LH and FSH basal concentrations should have increased by factors of 4.5 and 2, respectively. TRH test (200 µg i.v.): normal values for TSH, 0.5–6, 14±7 and <8 mU/l for basal, peak, and 120-min post-TRH. Inhibin B, normal values in young adults 135–350 pg/ml; Free T4 (FT4), normal values 11–21 pmol/l; Prolactin, normal values <20 ng/ml; HbA1c, normal values<5.8%. Insulin concentrations in an intravenous glucose tolerance test (IGTT) at T1+T3 min after glucose administration; normal values >40 mU/l. C-peptide, normal basal values: 0.3–1.3 nmol/l.

The mother experienced menarche at the age of 13 y and her first spontaneous pregnancy occurred at 20 y. The father reported normal pubertal development. He developed glucose intolerance by the age of 46 y. Both parents were of normal height, at 160 and 175 cm.

### Characterization of the *DMXL2* Mutation

Assuming a fully penetrant recessive transmission model with a disease allele frequency of 0.00001, two candidate regions were identified on chromosomes 13 and 15, with a LOD score of 2.5 (Figure 1B). These two regions harbored 164 and 341 genes. High-throughput sequencing of all exons of both candidate regions in the DNA of the proband and his mother revealed an in-frame deletion of 15 nucleotides (c.5824_5838del) in exon 24 of *DMXL2*, which encodes rabconnectin-3α (Rbcn-3α) (Figure 1C and Figure S1). This deletion results in the deletion of five amino-acid residues SDGNG (p.1942_1946del) (Figure 1C). Both parents and one unaffected sister were heterozygous for the deletion; one unaffected sister did not carry the deletion (Figure S1). This pattern of familial segregation is consistent with recessive transmission. The Ser1942 residue is highly conserved between species, whereas the other four residues are not well conserved. We suspected that the 15-nucleotide deletion in *DMXL2* might modify the level of expression of this gene [22]. In fact, quantitative RT-PCR showed that there was significantly less *DMXL2* mRNA in homozygous c.5824_5838del patients than in heterozygous or wild-type (WT) family members and unrelated controls (Figure 1D). These results suggested that the c.5824_5838del probably exerted its pathogenic effects by decreasing the expression of *DMXL2*. We tested this hypothesis by searching for *DMXL2* mutations in 10 cases of HH with a similar phenotype. No *DMXL2* mutation was found in any of these patients. We therefore carried out studies in rodents, to confirm the link between the decrease in *DMXL2* expression and the phenotype.

### Expression of *Dmxl2* and Rbcn-3α in Rodent Brain and Pituitary

**Figure 2:**
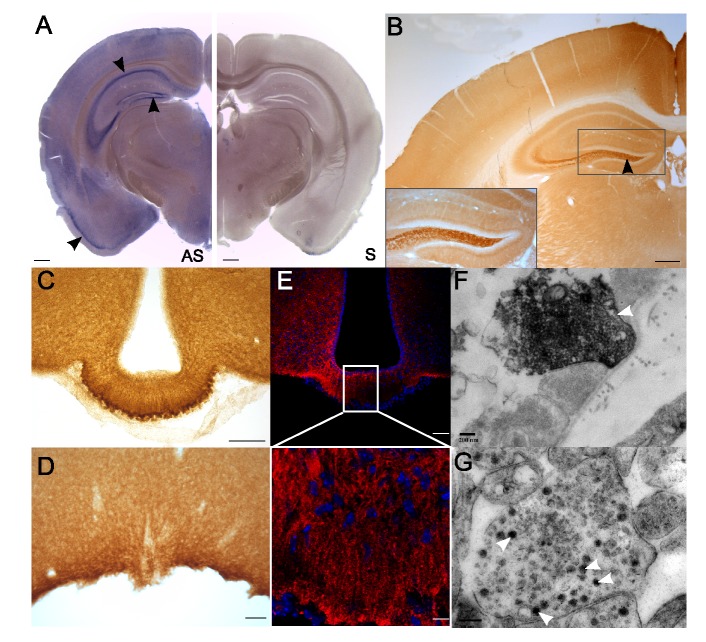
Rbcn-3α is expressed in exocytosis vesicles in the external layer of the median eminence. (A) ISH with a mouse *Dmxl2* antisense probe (AS) and a sense probe (S). (B) Immunolabeling with an antibody against Rbcn-3α revealed high levels of *Dmxl2*/Rbcn-3α expression in the dentate gyrus, the CA1 and CA3 regions of the hippocampus, and the cortex (black arrowheads). Scale bars, 200 µm. (C and D) Rbcn-3α was found to be strongly expressed in the external layer of the ME (C) and the OVLT (D). Scale bars, 100 µm. (E) Confocal analysis with an antibody against Rbcn-3α showed punctate staining in the median eminence and staining of the long processes extending from the cell bodies lining the third ventricle. (F and G) Rbcn-3α immunoreactivity was observed in small clear vesicles and LDCVs at the extremities of the axons in the ME (white arrow). Scale bar, 0.2 µm.

**Figure 3:**
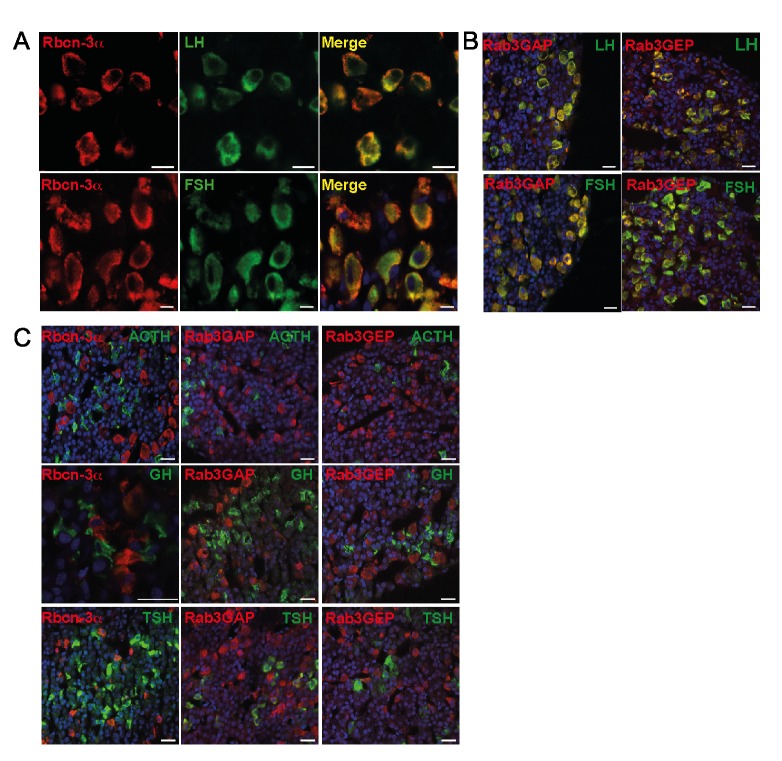
Rbcn-3α is specifically expressed in gonadotropes in the pituitary. (A) Double-immunostaining for Rbcn-3α and LH or FSH in the rat pituitary gland showed that Rbcn-3α was expressed in gonadotropes. Scale bars, 10 µm. (B) Double-immunostaining for Rab3GAP or Rab3GEP revealed that these two proteins were present in both LH- and FSH-expressing cells. (C) Immunostaining with antibodies against Rbcn-3α, Rab3GAP, or Rab3GEP and ACTH, TSH, and GH showed that Rbcn-3α, Rab3GAP, and Rab3GEP were not expressed in corticotropes, thyreotropes, and somatotropes. Scale bars, 40 µm. Red staining, antibodies against Rbcn-3α, Rab3GAP, and Rab3GEP. Green staining, antibodies against pituitary hormones.

Rbcn-3α is a synaptic protein that interacts closely with WDR7 [23] and may serve as a scaffold protein for both Rab3-GEP and Rab3-GAP on synaptic vesicles [23],[24]. Rbcn-3α has also been implicated in the regulation of Notch signaling, through the modulation of v-ATPAse activity in mammalian and insect cells [25]. We therefore analyzed the expression of *Dmxl2* and Rbcn-3α in both the brain and pituitary in rodents. Both *Dmxl2* mRNA and Rbcn-3α were detected in the hippocampus, dentate gyrus, hypothalamus, pyriform cortex (Figure 2A–E), and the granular and molecular layers of the cerebellum of adult mice (Figure S2). In the hypothalamus, immunostaining for Rbcn-3α was observed in the arcuate nucleus (ARC), the median eminence (ME), the organum vasculosum of the lamina terminalis (OVLT), (Figure 2C,D), and the subfornical organ (SFO), the subcommissural organ, and the suprachiasmatic nucleus (SCN) (Figure S2). The external layer of the ME displayed a punctate pattern of immunostaining for Rbcn-3α (Figure 2E). Staining was also observed in the cell bodies lining the third ventricle and in the long processes extending from these cell bodies toward the external layer of the ME (Figure 2E). Rbcn-3α was detected in small clear vesicles (Figure 2F) and in large dense core vesicles (LDCVs) (Figure 2G). This pattern of staining strongly suggests that both tanycytes and hypothalamic neurosecretory neurons express Rbcn-3α. In the anterior pituitary of adult rats, Rbcn-3α immunostaining was observed only in cells producing LH and FSH (Figure 3A), the pattern observed being similar to that for Rab3-GEP and Rab3-GAP (Figure 3B). None of these three proteins colocalized with ACTH, TSH, or GH (Figure 3C).

### In Vivo Investigation of *Dmxl2* Deletion in Mice

We then investigated whether a decrease in *Dmxl2* expression in mice could reproduce the phenotype observed in the human patients. For this purpose, we used a targeted gene-trapping vector to delete *Dmxl2* in mice [26]. In this strategy, the recombinant allele (tm1a) contained an IRES:LacZ trapping cassette and a floxed promoter-driven neo cassette in intron 6 and two loxP sites, one at either end of exon 7 of *Dmxl2* (Figure S3). The tm1a allele encodes a truncated form of Rbcn-3α. *Dmxl2*-heterozygous hypomorphic mice (*Dmxl2* ^tm1a/wt^) displayed no obvious morphological, growth deficit (Table S1). *Dmxl2* ^tm1a/wt^ mice were fertile. A tendency for a slightly later vaginal opening (VO), an important external indicator of puberty onset, and a longer time between VO and first estrus, were observed in *Dmxl2^tm1a/wt^* mice, but these differences were not significant (Figure S4). Although fertile, these mice produced no homozygous offspring (*Dmxl2* ^tm1a/tm1a^) when crossed (Table S2).

**Figure 4:**
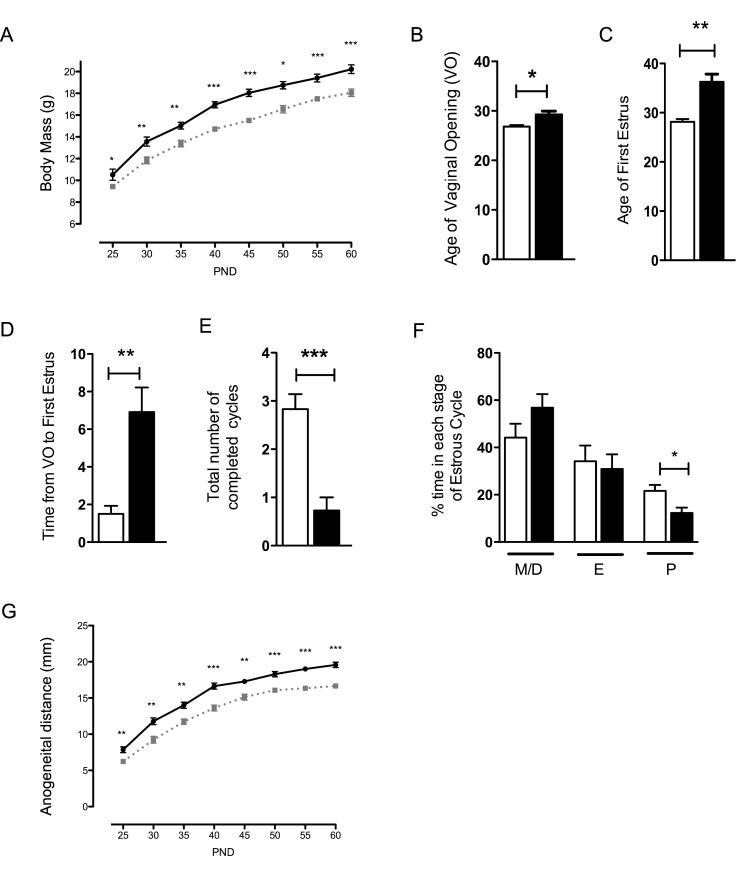
Female *Nes-cre;Dmxl2* ^–/wt^ mice displayed delayed puberty and were infertile. (A) Postnatal growth curve of female *nes-Cre*;*Dmxl2* ^–/wt^ mice (black line, *Dmxl2^lox/wt^*; hatched gray line, *nes-Cre*;*Dmxl2* ^–/wt^). (B and C) VO and first estrus occurred significantly later in female *nes-Cre*;*Dmxl2* ^–/wt^ mice than in their WT littermates. (D) The interval from VO to first estrus was significantly longer in female *nes-Cre*;*Dmxl2* ^–/wt^ mice than in WT mice, suggesting a defect in maturation of the HPG axis. (E) Very few complete estrous cycles were observed in female *nes-Cre*;*Dmxl2* ^–/wt^ mice. (F) Female *nes-Cre*;*Dmxl2* ^–/wt^ spent less time in high estradiol concentration (M, miestrus; E, estrus; D, diestrus; P, proestrus). (G) A significant difference in AGD was observed between male *nes-Cre*;*Dmxl2* ^–/wt^ mice and their WT littermates. Black line, *Dmxl2^lox/wt^*; hatched gray line, *nes-Cre*;*Dmxl2* ^-/wt^. White bars, *Dmxl2^lox/wt^*. Black bars, *nes-Cre*;*Dmxl2* ^–/wt^. Numerical data used to generate graphs 4B, 4C, 4D, 4E, 4F, or graphs 4A, 4G may be found in Table S5 and Table S6, respectively. Error bars are SEM. *p<0.05, **p<0.01, ***p<0.001.

As the gene-trapping method may generate a hypomorphic allele [27],[28] and we expected the severity of the phenotype to be correlated with *Dmxl2* expression levels in neurons, we investigated whether a mouse line with a complete KO of *Dmxl2* in neurons had a more severe phenotype. We crossed *Dmxl2* ^tm1a/wt^ mice with WT mice expressing the FLIP (flp) recombinase, which recognizes the FLP recombinase target (FRT) sequence flanking the β-galactosidase-neomycin cassette in the tm1a allele. This led to the production of mice bearing two WT alleles, with two loxP sites flanking exon 7 of *Dmxl2* (*Dmxl2* ^lox/lox^). These *Dmxl2* ^lox/lox^ mice were then crossed with mice expressing the Cre-recombinase under the control of the Nestin promoter (*nes-Cre*;*Dmxl2* ^wt/wt^) [29], to obtain mice bearing a *Dmxl2* allele from which exon 7 was deleted in nestin-expressing cells (*nes-Cre*;*Dmxl2* ^–/wt^) (Figure S3). Adult *nes-Cre*;*Dmxl2* ^–/wt^ mice displayed no obvious morphological defects. Hypothalamic *Dmxl2* mRNA levels were 50% lower in *nes-Cre;Dmxl2^–/wt^* than in *Dmxl2^lox/wt^* mice (Figure S3). Female *nes-Cre*;*Dmxl2* ^–/wt^ mice weighed less than WT mice (Figure 4A), whereas weight differences between males of these two genotypes were only observed from PND 30 to PND 40 (Figure S5). We first analyzed reproductive function. VO and first estrus occurred significantly later in female *nes-Cre;Dmxl2* ^–/wt^ mice than in their WT littermates (Figure 4B and 4C). The time from VO to first estrus was significantly longer in *nes-Cre*;*Dmxl2* ^–/wt^ mice (Figure 4D). Very few *nes-Cre*;*Dmxl2* ^–/wt^ females completed a full estrous cycle over a 20-d period (Figure 4E), and they spent less time in high estradiol levels (Figure 4F). In *nes-Cre*;*Dmxl2* ^–/wt^ males, the anogenital distance (AGD) was significantly shorter than that in their WT littermates, providing an external indicator of low testosterone concentration (Figure 4G). Fertility was assessed over a period of 3 mo. The female *nes-Cre*;*Dmxl2* ^–/wt^ mice had very poor fertility, producing few, if any litters, and the *nes-Cre*;*Dmxl2* ^–/wt^ males were subfertile (Table S3).

**Figure 5:**
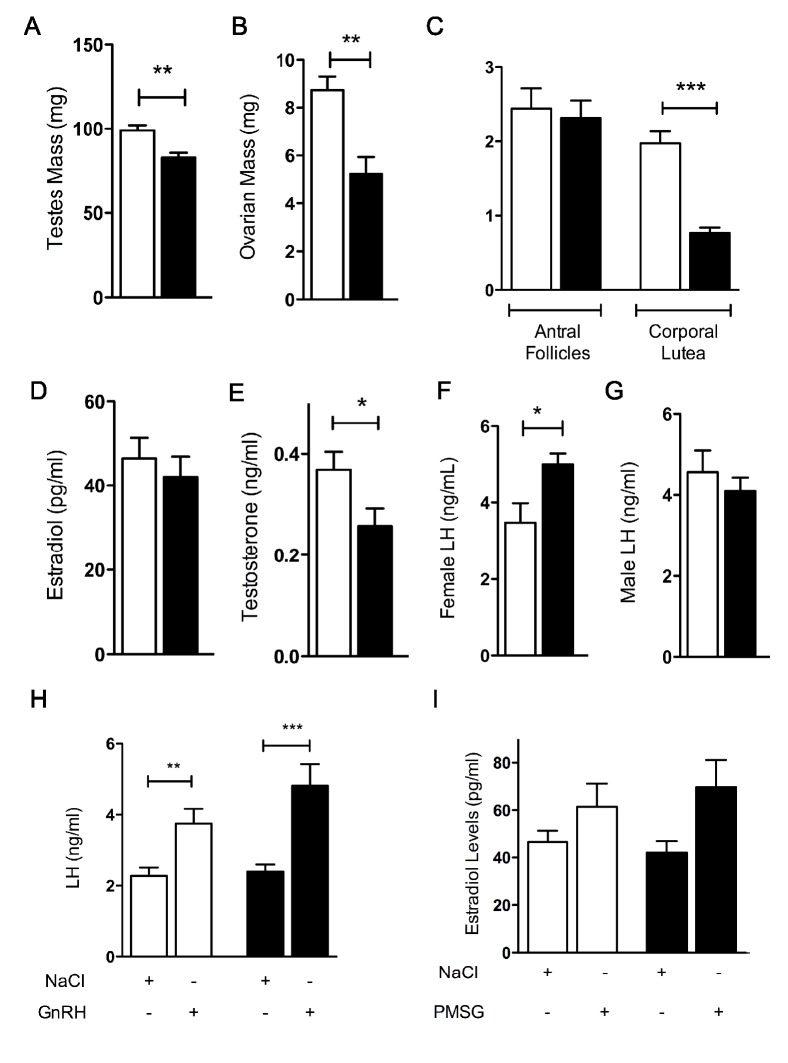
*nes-Cre*;*Dmxl2* ^–/wt^ mice displayed a partial gonadotropin deficiency. (A and B) Weights of testes and ovaries were low in *nes-Cre*;*Dmxl2* ^–/wt^ mice. (C) Histological analysis of ovaries showed a normal number of antral follicles but very few corpora lutea in female *nes-Cre*;*Dmxl2* ^–/wt^ mice. (D) Estradiol concentrations were normal in female *nes-Cre*;*Dmxl2* ^–/wt^ mice. (E) Plasma testosterone concentration was low in male *nes-Cre*;*Dmxl2* ^–/wt^ mice. (F) Plasma LH concentrations were moderately high in female *nes-Cre*;*Dmxl2* ^–/wt^ mice. (G) Despite their lower testosterone concentrations, male *nes-Cre*;*Dmxl2* ^–/wt^ mice had normal plasma LH concentrations. (H) The GnRH-induced increase in LH concentration was normal in *nes-Cre*;*Dmxl2* ^–/wt^ mice. (I) The administration of PMSG to young mice induced a normal increase in estradiol concentration in *nes-Cre*;*Dmxl2* ^–/wt^ mice, similar to that observed in their WT littermates (asterisks indicate significant differences: * *p*<0.05, ** *p*<0.001; ***p<0.0001). Error bars: SEM. P, postnatal day. White bars, *Dmxl2^lox/wt^*; black bars, *nes-Cre*;*Dmxl2* ^–/wt^. Numerical data used to generate these graphs may be found in Table S5.

Phenotypic analyses were then pursued to characterize the fertility problems observed in *nes-Cre*;*Dmxl2* ^–/wt^ mice. The weights of testes and ovaries were low in *nes-Cre*;*Dmxl2* ^–/wt^ mice (Figure 5A and 5B). A histological analysis of ovaries in female *nes-Cre;Dmxl2* ^–/wt^ mice revealed the presence of normal numbers of antral follicles associated with significantly fewer corpora lutea than would normally be expected (Figure 5C). Estradiol concentrations were normal in *nes-Cre*;*Dmxl2* ^–/wt^ females (Figure 5D), whereas testosterone concentrations were significantly lower in *nes-Cre*;*Dmxl2* ^–/wt^ males than in their WT littermates (Figure 5E). LH concentrations were moderately, but significantly, higher in *nes-Cre*;*Dmxl2* ^–/wt^ females than in WT females (Figure 5F), whereas no difference in LH concentration was observed between *nes-Cre*;*Dmxl2* ^–/wt^ and WT males (Figure 5G).

The normal concentrations of LH observed despite the low concentration of testosterone in male *nes-Cre*;*Dmxl2* ^–/wt^ mice was indicative of a partial CHH due to a hypothalamic defect. Consistent with this, *nes-Cre*;*Dmxl2* ^–/wt^ mice displayed normal increases in LH concentration following the intraperitoneal injection of GnRH (Figure 5H). The normal response to PMSG injection of ovaries from *nes-Cre*;*Dmxl2* ^–/wt^ mice confirmed that the very low rate of ovulation in *nes-Cre*;*Dmxl2* ^–/wt^ females was related to a defect in the hypothalamo–pituitary control of the gonadotropic axis (Figure 5I). The delayed puberty and infertility observed in *nes-Cre*;*Dmxl2* ^–/wt^ mice was therefore due largely to a hypothalamic defect.

**Figure 6:**
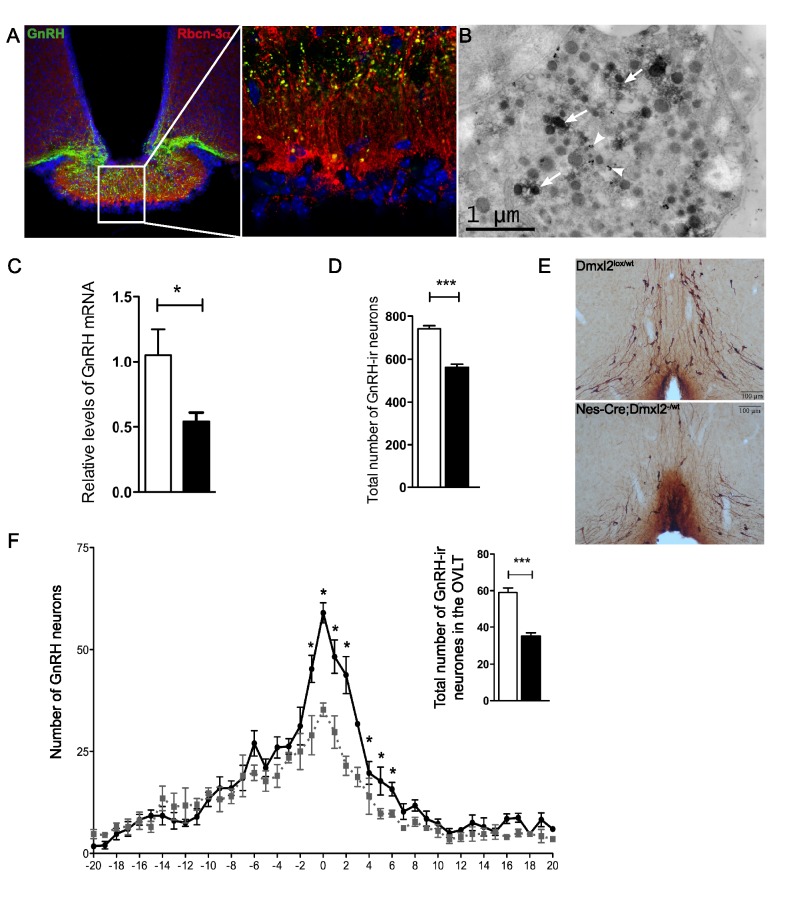
Hypothalamic GnRH mRNA and GnRH-IR neuron levels are lower in the hypothalamus of *nes-Cre*;*Dmxl2* ^–/wt^ mice. (A) Rbcn-3α is expressed in GnRH neurons in the median eminence. (B) Rbcn-3α is located in small clear vesicles and in LDCVs in GnRH neurons. White arrows indicate Rbcn-3α DAB staining; white arrow heads indicate GnRH nanogold staining. (C) *GnRH1* mRNA levels relative to RNA18S were lower in the hypothalamus of *nes-Cre*;*Dmxl2* ^–/wt^ male mice than in WT mice. (D) The total number of GnRH-ir neurons in the brain was lower in *nes-Cre*;*Dmxl2* ^–/wt^ male mice than in WT mice. (E) GnRH immunostaining in the OVLTs in *Dmxl2* ^lox/wt^ and *nes-Cre*;*Dmxl2* ^–/wt^ male mice. (F) An analysis of the rostral–caudal distribution of GnRH-ir neurons in the hypothalamus revealed that *nes-Cre*;*Dmxl2* ^–/wt^ male mice had fewer GnRH-ir cell bodies in the OVLT (see inset) than their WT littermates. * *p*<0.05, *** *p*<0.0001. White bars, *Dmxl2^lox/wt^*; black bars, *nes-Cre*;*Dmxl2* ^–/wt^. Numerical data used to generate graphs 6C, 6D, 6F, and 6F inset may be found in Table S5.

We characterized the hypothalamic defect in *nes-Cre*;*Dmxl2* ^–/wt^ mice, by determining whether Rbcn-3α was produced in GnRH neurons. Punctate Rbcn-3α staining was colocalized with GnRH in the median and external layers of the ME (Figure 6A). Electron microscopy confirmed that Rbcn-3α was present in GnRH neurons (Figure 6B). We investigated whether the gonadotropin deficiency observed in *nes-Cre*;*Dmxl2* ^–/wt^ mice was due to GnRH deficiency, by quantifying GnRH mRNA levels in the hypothalamus of *nes-Cre*;*Dmxl2* ^–/wt^ mice and comparing them with those of WT mice. GnRH mRNA levels were significantly lower in *nes-Cre*;*Dmxl2* ^–/wt^ mice than in WT mice (Figure 6C). This decrease was specific to GnRH, as the amounts of mRNA for other hypothalamic-releasing hormones were normal (Figure S6). The lower levels of GnRH mRNA were associated with the presence of fewer GnRH-ir neurons in the hypothalamus of *nes-Cre*;*Dmxl2* ^–/wt^ mice than in WT littermates (Figure 6D). An analysis of the rostral–caudal distribution of GnRH-ir neurons from the diagonal band of Broca to the ME showed that there were significantly fewer GnRH-ir neurons in the OVLT, this loss of neurons not being compensated by an increase in the number of GnRH-immunoreactive (GnRH-ir) neurons in the rostral or caudal parts of the hypothalamus (Figure 6E and 6F). The poor fertility observed in *nes-Cre*;*Dmxl2* ^–/wt^ mice was therefore associated with the presence of fewer GnRH-ir neurons in the hypothalamus.

As patients with the c.5824_5838del of *DMXL2* displayed ataxia and mental disability, we investigated whether *Dmxl2^lox/wt^* or *nes-Cre*;*Dmxl2* ^–/wt^ mice displayed neurological defects. We analyzed the gait and exploratory behavior of these mice in an open-field system [30]. No significant difference of the gait between *Dmxl2^tm1a/wt^* mice and their WT littermates was observed. However, *nes-Cre*;*Dmxl2* ^–/wt^ mice displayed significantly higher levels of exploratory behavior than their WT littermates (Figure S7).

### Rbcn-3α Is Involved in the Regulated Secretion of Insulin

**Figure 7:**
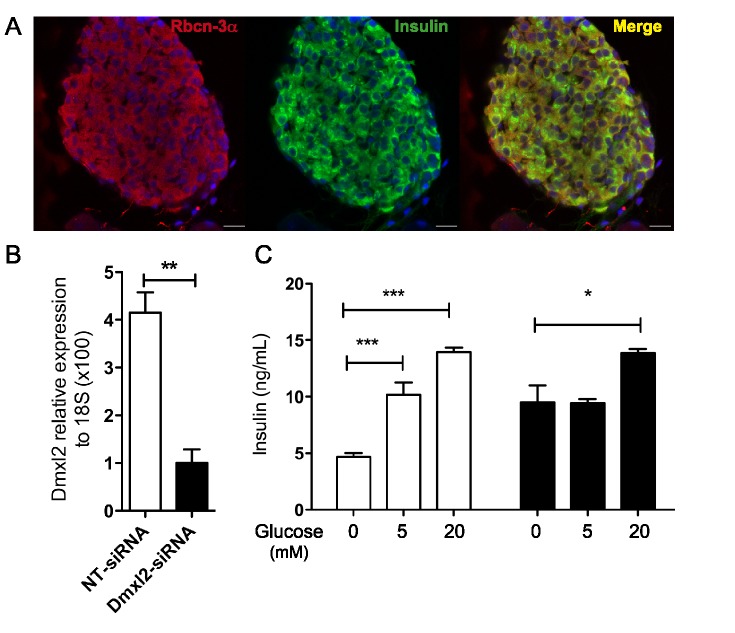
*Dmxl2* knockdown in INS-1E cells decreases glucose-induced insulin release. (A) Rbcn-3α is expressed in insulin-secreting cells in the islets of Langerhans in the mouse pancreas. (B) Seventy-two hours after the transfection of INS-1E cells with *Dmxl2-*siRNA, *Dmxl2* mRNA levels had decreased by 75% (error bars: SEM; from three independent experiments). (C) Seventy-two hours after siRNA transfection, the release of insulin was evaluated by quantifying insulin concentrations in cell supernatants. In the absence of glucose, insulin concentrations in *Dmxl2*-siRNA–transfected cells were double those in cells transfected with NT siRNA (NT-siRNA). In the presence of various concentrations of glucose (5 and 20 mM), *Dmxl2*-siRNA–transfected cells displayed only a small increase in insulin release, at a glucose concentration of 20 mM only, whereas a 2- to 3-fold increase was observed with NT siRNA-transfected cells. Error bars: SEM from one representative experiment performed twice, in hextuplicate. * *p*<0.05, ** *p*<0.001, *** *p*<0.0001. Numerical data used to generate graphs 7B and 7C may be found in Table S5.

Severe abnormal glucoregulation, consistent with impaired insulin secretion, was observed in the three affected brothers. Rbcn-3α forms a complex with regulators of Rab3 GTPase, and this complex is involved in the docking and priming of exocytosis vesicles [31]. Insulin-secreting pancreatic islet β cells express many proteins involved in the exocytosis of synaptic vesicles [32],[33]. In fact, Rbcn-3α was found to be present in pancreatic β cells (Figure 7A). We therefore hypothesized that Rbcn-3α might be involved in the regulated secretion of insulin. We tested this hypothesis, by using siRNA to down-regulate *Dmxl2* expression in an insulin-secreting cell line (INS-1E). We observed a significant decrease in *Dmxl2* mRNA levels, to 25% those in nontarget (NT) siRNA-transfected cells, 72 h after the transfection of INS-1E cells with the *Dmxl2* siRNA (Figure 7B). The knockdown of *Dmxl2* expression with *Dmxl2* siRNA resulted in significantly higher levels of basal insulin secretion than for NT siRNA-transfected cells (Figure 7C). By contrast to what was observed for NT siRNA-transfected cells, the incubation of *Dmxl2* siRNA-transfected cells with 5 mM glucose did not increase insulin secretion, and only a slight increase in insulin secretion was observed after incubation with 20 mM glucose (Figure 7C). These in vitro experiments demonstrate the involvement of Rbcn-3α in the control of regulated insulin secretion.

## Discussion

In this study, we found that a low level of *DMXL2* haploinsufficiency caused a previously unknown complex phenotype in humans. The Warburg Micro and Martsolf syndromes were considered the most relevant syndromes for comparison with the phenotype reported here, as these syndromes are caused by mutations affecting the catalytic (*RAB3GAP1*, p130) and noncatalytic subunits (*RAB3GAP2*, p150) of RAB3GAP [18],[19], two components of the protein complex containing Rbcn-3α. Hypogonadotropic hypogonadism, mental retardation, and demyelinating peripheral neuropathy have been reported in patients with *RAB3GAP1* or *RAB3GAP2* mutations [34], but the bilateral cataracts, microphthalmia, and microcornea systematically found in patients with the Warburg Micro and Martsolf syndromes were not observed in this family. Furthermore, insulin-dependent diabetes mellitus has not been reported in these syndromes. The phenotype observed in this family was therefore different from those of the Warburg Micro and Martsolf syndromes.

Mice expressing a conditional heterozygous null allele of *Dmlx2* in neurons have very low fertility. This finding contrasts with the observed phenotype of mice expressing a hypomorphic *Dmxl2* allele and with findings for the family studied here, in which the two c.5824_5838del-heterozygous parents were clearly fertile. We suspect that the severity of the phenotype is highly associated to the neuronal expression level of *Dmxl2*. The level of Rbcn-3α expression in the neurons of *nes-Cre*;*Dmxl2* ^–/wt^ mice was found to be 50% that in WT animals, whereas *Dmxl2* expression in the neurons of *Dmxl2^tm1a/wt^* mice was thought to be more than 50% that observed in WT mice. *DMXL2* mRNA levels were about 30% normal values in subjects homozygous for c.5824_5838del, whereas they were about 60% normal values in subjects heterozygous for this deletion (see Figure 1). Furthermore, we were unable to obtain viable *Dmxl2^tm1a/tm1a^* mice. These mice died during embryonic development, indicating a requirement of Rbcn-3α for normal development in mice. This correlation between *DMXL2* expression level and the severity of the neurological and neuroendocrinological phenotypes in both mice and humans is consistent with a probable synaptic function for Rbcn-3α. The phenotypic discrepancy between mice and humans may also result from the interactions of different partners with Rbcn-3α. It may also reflect the fact that the threshold of the neuronal expression level of *Dmxl2* causing a phenotype may be species-specific.

An investigation of gonadotropic axis function in adult *nes-Cre*;*Dmxl2* ^–/wt^ mice showed that the partial gonadotropic deficiency was associated with the presence of a smaller total number of GnRH-ir neurons. Multiple mechanisms may be involved, such as a defect of GnRH neuron differentiation in the OP, migratory failure, or a defect in the maturation and postnatal survival of these GnRH neurons. The latter follow a unique migratory route, emerging in the OP at E10.5 and then entering the forebrain at E.13.5, to migrate along olfactory/vomeronasal nerve axons before turning toward the hypothalamus, where they are dispersed in the preoptic area (POA) and extend their axons by E16.5 [35]. Many of the factors controlling GnRH neuron migration also influence or control the development of the olfactory system [35]. Olfactory bulbs developed normally in *nes-Cre*;*Dmxl2* ^–/wt^ mice, demonstrating that the smaller number of GnRH neurons in the POA region of *nes-Cre*;*Dmxl2* ^–/wt^ adult mice was not associated with a defect of olfactory/vomeronasal nerve axon development. This result is consistent with the absence of anosmia and olfactory bulb agenesis in the patients with *DMXL2* mutations. Furthermore, the analysis in adult mice showed that the migration of GnRH neurons was not halted in the forebrain. Preliminary data suggested that adult nes-Cre;Dmxl2^–/wt^ mice had a GnRH neuron morphology in the rostral POA that was more complex than the normal unipolar/bipolar morphology displayed by their WT littermates. This region contains the GnRH neuron population of the greatest importance for the onset of puberty and fertility. This complex morphology may indicate that GnRH neurons did not progress toward a mature form. This led us to suspect that there might be a defect in the postnatal development of GnRH neurons in *nes-Cre*;*Dmxl2* ^–/wt^ mice. It is interesting to note that Rbcn-3α was recently shown to be required for neural crest cell migration and cell fate determination by controlling the Wnt signaling pathway [36].

The observation that siRNA-induced down-regulation of *Dmxl2* expression led to an increase in spontaneous insulin release and a disruption of glucose-induced insulin release in INS-1E cells is consistent with a role for Rbcn-3α in controlling the secretion of insulin in vivo. The episodes of hypoglycemia during childhood in the human patients, associated with the lack of a blockade of insulin release, may be accounted by a decrease in the inhibition of basal insulin release by Rbcn-3α. By contrast, the occurrence of insulin-dependent diabetes mellitus during adolescence in the affected patients indicates an inability of the pancreas to respond to the increase in insulin requirement at puberty [37]. This role of Rbcn-3α in controlling insulin release is consistent with the recent report of an association between the MAP kinase-activating death domain (MADD) protein locus, also known as RAB3GEP, with fasting glycemia and plasma proinsulin concentrations [38],[39]. Rab27a is a monomeric GTPase protein very similar to Rab3. Rab27a mediates glucose-specific signals for the exocytosis of insulin vesicles in the pancreas [40]. Further investigations are required to confirm the role of Rbcn-3α in insulin release in vivo and to determine whether this protein may also act via the Rab27 pathway.

As the cellular machinery controlling vesicle exocytosis is similar in pancreatic β cells and neurons [32],[33], we can assume that Rbcn-3α is involved in neurosecretion. Rbcn-3α may exert its neurosecretory functions by controlling the “on/off” activity of Rab3a [41],[42]. However, the severity of the phenotypes observed in this family and in patients with *RAB3GAP1* and *RAB3GAP2* mutations [18],[19] contrast with the mild deficiency of Ca^2+^-triggered neurotransmitter release caused by *Rab3a* deletion [43]. Other vesicle exocytosis steps may thus be impaired when *Dmxl2* expression levels are low in neurons [44]–[46]. The neurotransmitter glutamate is involved in the regulation of GnRH secretion [3]. Glutamate release is controlled by the amount of active Rab3a [42], suggesting that glutamate-induced GnRH neuron activation may be impaired in *nes-Cre*;*Dmxl2* ^–/wt^ mice. This possible dysfunction of the glutamatergic input in GnRH neurons may be responsible for the loss of GnRH neurons in *nes-Cre*;*Dmxl2* ^–/wt^ mice. Defective V-ATPase–mediated acidification of exocytosis vesicles may also contribute to the phenotype [47]. The expression of Rbcn-3α, Rab3-GEP, and Rab3-GAP in gonadotropes only suggests that these three proteins may specifically regulate the secretion of LH and FSH. A dual defect in hypothalamic neuropeptide and gonadotropin secretion may thus contribute to the gonadotropic axis deficiency observed in the studied family.

We found in this study that low levels of *Dmxl2* expression caused a complex neurological and endocrine phenotype in humans. Low levels of *Dmxl2* expression in neuronal cells of mice lead to a partial gonadotropic axis deficiency, resulting in a very low fertility, due to a loss of GnRH neurons in the hypothalamus. Our results strongly suggest that the loss of these neurons is due to a dysfunction of peptide/neurotransmitter release by GnRH neurons itself or the neurons innervating GnRH neurons. This association of a low level of expression of a vesicular protein and the loss of GnRH neurons constitutes a new mechanism of GnRH deficiency. This study opens up new possibilities for characterizing the pathogenic mechanisms of GnRH deficiency and the neuronal control of the GnRH network.

## Materials and Methods

This study was approved by the institutional review boards of Robert Debré Hospital and Paris Diderot University. All participants provided informed written consent for genetic analyses. Animals were deeply anesthetized by pentobarbital injections. The number of the ethical approval was 2012-15-676-0099. It was delivered by the IFR Claude Bernard.

### Genome Mapping and Characterization of the Genetic Abnormality

DNA was extracted from peripheral blood lymphocytes and genotyped with an Affymetrix 250k Nsp array, according to the manufacturer's instructions. Linkage analysis was performed by classical techniques [48],[49]. Call rates were >98%, and multipoint LOD scores were calculated with Allegro-v1.2c [49], after the random selection of one informative SNP every 500 kb, following data quality control with Alohomora-v0.33 software [48]. A fully penetrant recessive transmission model was assumed, with a disease allele frequency of 0.00001. Two genomic regions were found to have LOD scores of 2.5, corresponding to the maximum possible LOD score for this family. High-throughput sequencing of the two candidate regions was performed at the “Centre National de Génotypage” in Evry, France. Sanger sequencing of *DMXL2* exons was performed for all family members (Figure S1) and for 10 additional patients. Primer sequences are available upon request.

### Expression Analysis by Quantitative RT-PCR

Total RNA was extracted from blood lymphocytes from the patients, with a QiaAMP RNA Blood Mini Kit (Qiagen), according to the manufacturer's instructions. Total RNA was extracted from mouse tissues with Trizol (Life Technologies). The first-strand cDNA was synthesized with the Superscript II first-strand synthesis kit (Invitrogen), with random primers or oligodT. Human *DMXL2* and 18S mRNA levels in the patients' lymphocytes were quantified in specific *DMXL2* and RNA 18S *Taq*man gene expression assays on a 7300 Real-Time PCR System (Applied Biosystems). Reverse transcription–quantitative PCR (RT-qPCR) analysis of gene expression in INS-1E cells or in mouse tissues was performed with the SyberGreen MasterMix (BioRad, Hercules, CA), in a CFX 384 Real-Time PCR Detection System (BioRad). The primer sequences used are available upon request. Gene expression levels are expressed relative to those of *RNA 18S* or *GAPDH*, as follows: 100×2(e^−ΔCt*Gene*^/e^−ΔCt*Housekeeping Gene*^).

### In Situ Hybridization (ISH)

We analyzed *Dmxl2* expression in the brain by performing ISH on C57Bl6 mouse brain, with a probe generated from a DNA template obtained by PCR. A digoxigenin UTP-labeled *Dmxl2* probe was synthesized by generating a DNA template by PCR, with the Expand High-Fidelity PCR system (Roche Diagnostics) and a pair of primers binding between nucleotides 222 and 1068 of the mouse *Dmxl2* cDNA sequence (NCBI Reference Sequence NM_172771.2) and carrying the SP6 and T7 promoter sequences. The PCR product was used as the DNA template for the transcription of digoxigenin-UTP probes with an SP6 and T7 DIG RNA labeling kit (Roche Diagnostics).

ISH was carried out on 200-µm mouse brain sections. Mouse brains were embedded in a 5% agarose/BSA preparation and sectioned with a vibratome (Leica VT 1000 S). Sections were dehydrated by passage through a graded series of ethanol solutions and were stored frozen at −20°C. Sections were rehydrated and ISH was carried out as previously described [50]. Sections were mounted on glass slides, in 80% glycerol in PBS, and images were captured under a microscope (Leica DM IRB), at a magnification of 1.6×. Half-brain images were reconstituted from several images of the same section, taken at the same time and under the same conditions.

### Immunohistochemistry (IHC)

For the analysis of Rbcn-3α expression in the rodent pituitary and brain, tissues were prepared from C57BL6 mice and/or WKY rats, as previously described [51]. The rodents were deeply anesthetized by pentobarbital injections and perfused transcardially with 4% paraformaldehyde in 0.1 M phosphate-buffered saline (PBS), pH 7.4. Brains were removed, cryoprotected in 30% sucrose, frozen in liquid isopentane at −45°C, and stored at −80°C until sectioning. We cut 30-µm coronal sections on a cryostat, and the free-floating sections were processed for IHC. For immunofluorescence, sections were incubated with 5% normal donkey serum (NDS) and 0.4% Triton X-100 in PBS (PBS-T) for 45 min. Sections were then incubated with primary antibodies in 1% donkey serum in PBS-T, for 48 h at 4°C (Table S4). Sections were rinsed several times with PBS and then incubated with a fluorescent secondary antibody for 1 h at room temperature (RT). They were then washed three more times in PBS, counterstained with TO-PRO-3 (1/500, Invitrogen), rinsed in PBS, mounted on Superfrost Plus slides (Microm Microtech) in Fluoromount (Southern Biotech), and covered with a coverslip.

For immunoperoxidase staining, the same procedure was followed, except that the sections were incubated with a biotinylated secondary antibody for 90 min at RT. The sections were incubated with 1% H_2_O_2_ in PBS for 10 min, rinsed in PBS-T, and labeling was detected with the Vectastain ABC kit (Vector Laboratories, Burlingame). Sections were then mounted in Pertex resin.

The analysis of the GnRH neuron distribution was processed with the SW-1 antibody [52] at a dilution of 1∶3,000 in PBST (0.4%), for 48 h at 4°C, in the presence of 2% NDS and then as described above. The total number of GnRH-IR neurons was quantified in serial 40-µm coronal sections from the anterior preoptic region to the posterior hypothalamus in adult mice (PND 60), as previously described [53]. All slides were coded and scored blind. A neuron was scored as GnRH-IR only if it had a clearly stained cell body. We analyzed the distribution of GnRH-ir neurons further, designating the start of the OVLT as section 0. We numbered the 20 sections on either side of section 0 in the OVLT. This analysis did not include the regions in the immediate vicinity of the olfactory bulbs, but it did encompass most of the GnRH neuronal system of the mouse brain.

For double-labeling fluorescent IHC, sections were first rinsed in PBS and preincubated for 30 min in 5% NDS in PBS containing 0.3% Triton X-100. Sections were coincubated overnight at RT in ADD AbIs diluted in PBS containing 1% NDS and 0.3% Triton X-100. Sections were then rinsed in PBS and incubated for 1 h in GIVE THE AbIIs Alexa Fluor 488-conjugated donkey anti-rabbit IgG (1∶200; Molecular Probes) and Cy3-conjugated donkey anti-mouse IgG (1∶300; Jackson ImmunoResearch) diluted in PBS containing 3% NDS and 0.3% Triton X-100. Sections were then rinsed in PBS, counterstained with DAPI (1/4,000), mounted on glass slides, and coverslipped with Fluoromount-G solution (Southern Biotech) for confocal microscopic examination.

Immunofluorescent sections were analyzed using a Leica TCS SP8 confocal scanning system (Leica Microsystems) equipped with 488-nm Ar, 561-nm DPSS, and 633-nm HeNe lasers. Eight bit digital images were collected from a single optical plane using a 63× HC PL APO CS2 oil-immersion Leica objective (numerical aperture, 1.40) or a 40× HC PL APO CS2 oil-immersion Leica objective (numerical aperture, 1.30). For each optical section, double/triple-labelled images were acquired in sequential mode to avoid potential contamination by linkage-specific fluorescence emission cross-talk.

### Electron Microscopy

C57BL6 WT adult mice (PND 60) were anesthetized with 0.2 cm^3^ of 10% chloral hydrate and intracardially perfused with 100 ml of 4% PFA, 0.1% glutaraldehyde in 0.1 M phosphate buffer (pH 7.4) on ice. Brains were postfixed by overnight incubation in 4% PFA at 4°C. The following day, the hypothalamus was dissected and cut into 200-µm sections in ice-cold PBS. These sections were then processed for pre-embedding IHC with an antibody against rabconnectin-3 [54]. For this purpose, sections were immersed for 20 min in 50 mM ammonium chloride in PBS, rinsed, and incubated overnight at 4°C with rabbit anti–rabconnectin-3α antibody (1∶500) in PBS containing 0.1% gelatin (PBSg). Antibody binding was detected by incubating sections with a goat anti-rabbit biotinylated antibody (1∶200) in PBS-gelatin for 4 h at RT and then with an avidin–peroxidase complex (1∶200) for 3 h (Elite Vectastain kit, Vector Laboratories). Finally, sections were incubated with 0.6% diaminobenzidine in Tris-buffered saline and then with Sigma Fast DAB. After staining, the region of interest, the ME, was further dissected and embedded in epoxy resin, for the cutting of semithin sections (0.5 µm), which were stained for visualization of the ME. Ultrathin sections (70 nm) of the ME were then cut and collected on 400-mesh formvar-coated nickel grids and examined with a Jeol JEM-100CXII electron microscope.

For dual labeling with Rbcn-3α and GnRH antibodies, the ME was carefully dissected out, rinsed in PBS, and incubated in 20% glycerol/20% sucrose in PBS for 3 h. ME sections were then placed carefully in liquid nitrogen for three freeze cycles, rinsed in NH_4_Cl, and washed in PBSg. ME sections were incubated overnight in mouse anti-GnRH antibody, at a dilution of 1∶200 in PBSg, at 4°C. The sections were then washed in PBSg, incubated with goat anti-mouse secondary antibody conjugated to gold particles (Aurion, Biovalley), at a dilution of 1∶100, for 4 h at RT (nanogold). Sections were washed in PBS. The nanogold particle signal was amplified with a BBI Amplification silver kit, for 7 min at RT, followed by gold toning. The sections were rinsed in PBS and incubated overnight at 4°C with rabbit anti–Rbcn-3α, at a dilution of 1∶500 in PBSg. Rbcn-3α staining was detected by incubating the sections with donkey anti-rabbit biotinylated antibody at a dilution of 1∶200 in PBSg at RT, rinsing in PBS, and then incubating with an avidin–biotin complex at a dilution of 1∶200, with the detection of DAB staining after postfixation (1% glutaraldehyde) with SigmaFast tablets (Sigma Aldrich). The DAB reaction was stopped with Tris-HCL, and the sections were washed in PBS. Sections were incubated with osmium tetraoxide for 1 h at 4°C and dehydrated for embedding in epoxy resin, flat embedding, and polymerization. Sections embedded in epoxy resin blocks were subsequently cut into semithin (0.5–2 µm) and ultrathin (70 nm) sections and analyzed by electron microscopy.

### In Vitro Analysis of Insulin Secretion

The role of Rbcn-3α in regulated insulin secretion was analyzed in the rat insulinoma-derived INS-1E cell line by knocking down *Dmxl2* expression with siRNAs directed against *Dmxl2*. Glucose-induced insulin release into the supernatant of *Dmxl2* siRNA-transfected cells was then compared with that from NT siRNA-transfected cells. The rat insulinoma-derived INS-1E cell line was kindly provided by Prof. Pierre Maechler and Prof. Claes Wollheim [55]. Cells were cultured in RPMI-1640 medium supplemented with 5% fetal calf serum. Twenty-four hours before transfection, cells were used to seed 96-well culture plates (BD Biosciences), in complete culture medium. They were then transfected with 100 nM siGENOME Smart Pool Rat LOC315676 siRNA against rat *Dmxl2* (Thermo Scientific) or 100 nM control siGENOME NT siRNA #1 (Thermo Scientific), in the presence of Dharmafect 2 transfection reagent (Thermo Scientific). The cells were incubated for 72 h, washed once in PBS, and used for total RNA extraction with Trizol reagent. Cells transfected with either *Dmxl2* or NT siRNA were incubated for 3 h with Krebs-Ringer bicarbonate buffer supplemented with 0.35% BSA (KRBH-BSA; Sigma Aldrich). They were then incubated in the same buffer with 0, 5, or 20 mM D(+) glucose monohydrate (Sigma Aldrich) for 90 min at 37°C, and insulin secretion was evaluated with the sensitive-range protocol of the HTRF insulin assay (Cisbio Bioassays).

### Generation of *Dmxl2* Hypomorphic and Nestin-cre/*Dmxl2* Mice

The gene targeting vector used to decrease the expression of *Dmxl2* in all cells included an additional acceptor splice site in intron 6, *LacZ* under the control of the endogenous *Dmxl2* promoter, a neomycin resistance (*neo^R^*) cassette under the control of the actin promoter, and *loxP* sites flanking the critical *Dmxl2* exon 7 (Figure S2). The vector (*Dmxl2* ^tm1a(EUCOMM)Wtsi^) was injected into JM8-F6 embryonic stem cells at the European Conditional Mouse Mutagenesis Program (EUCOMM). These cells were then injected into C57Bl/6N blastocysts at the Transgenic Animal Service (SEAT; Villejuif, France) to create chimeric mice, which were crossed to create *Dmxl2* hypomorphic (*Dmxl2* ^tm1a/wt^) mice. This mouse line was transferred to the INSERM U1141 animal facility, where the animals were housed under a 12 h light/12 h dark cycle and fed *ad libitum*. *Dmxl2* ^tm1a/wt^ mice were intercrossed to obtain *Dmxl2* ^wt/wt^ and *Dmxl2* ^tm1a/wt^ mice. However, we were unable to obtain any *Dmxl2* ^tm1a/tm1a^ mice due to embryonic lethality.

A conditional KO of *Dmxl2* was obtained by crossing *Dmxl2* ^tm1a/wt^ mice with FLIP (FLP) recombinase-expressing mice, to remove the β-galactosidase cassette and the neomycin resistance gene and to create *Dmxl2^lox/lox^* mice, which were then crossed with nes-Cre mice to obtain mice with a conditional deletion of *Dmxl2* in neurons and glial cells (nes-Cre;*Dmxl2^–/wt^*).

### Genotyping of Mice

Genomic DNA was obtained on postnatal day (PND) 21 (tail biopsy) and PND 60 (brain tissue), and each animal was genotyped by PCR. For detection of the *Dmxl2* GT cassette, two primers flanking the 5′ and 3′ regions of the GT cassette (*Dmxl2* 5′-arm and *Dmxl2*-3′arm) were used, together with another primer designed to bind within the 5′-arm of the GT cassette (*Dmxl2* LAR3) (www.knockoutmouse.org/about/eucomm). A separate PCR analysis of genomic DNA was used to investigate the presence of the *Nestin-Cre* allele.

### Phenotyping of *Dmxl2* KO Mice

The reproductive phenotype was assessed at 16:00 hours, at 5-d intervals, in male mice and for 20 consecutive days after weaning (PND 21) in females. In males, AGD was recorded from weaning (PND 21) to PND 60 (AGD). In females, three parameters were assessed: (1) age at VO, (2) age at first estrus, and (3) the interval between VO and first estrus. Estrous cyclicity was monitored daily after VO, at 16:00 hours, for 20 consecutive days, by vaginal lavage.

### GnRH Injections to Test Gonadotrope Responsiveness

Male mice underwent subcutaneous injections of 100, 200, 300, and 400 ng/kg GnRH in an initial trial experiment, to determine an appropriate submaximal dose for the LH response. A dose of 300 ng/kg was chosen as the submaximal dose for use in subsequent experiments. Five male mice of each genotype received 100 µl i.p. injections of 300 ng/kg GnRH (Sigma Aldrich) or vehicle (0.9% saline). Ten minutes after injection, mice were anesthetized rapidly by isoflurane inhalation and decapitated for trunk blood collection. Plasma was isolated at 4°C, after the centrifugation of blood samples at 3,000×*g* for 10 min. Plasma samples were then stored at −20°C until use.

### PMSG Test of Ovarian Responsiveness

Five young (PND21-PND23) female mice of each genotype underwent i.p. injections of 0.4 IU/g PMSG (Sigma Aldrich) or vehicle (0.9% saline). They were anesthetized with isoflurane vapor 48 h later and decapitated for trunk blood isolation. Plasma was isolated at 4°C, by centrifugation at 3,000×*g* for 10 min.

### Hormone Assays

Trunk blood from adult PND60 mice was collected into heparin, after decapitation at 16:00. The blood was immediately placed on ice and was then centrifuged at 4°C and 3,000×*g* for 10 min. Plasma was subsequently harvested and stored at −20°C until analysis. Plasma luteinizing hormone concentrations were assessed by a radioimmunoassay (RIA) [56]. Plasma estradiol concentrations were determined with an estradiol enzyme immunoassay (EIA) kit (Caymen Chemicals, 582251). The 80% B/B0 detection limit for this kit is approximately 19 pg/ml, with a sensitivity of 129 pg/ml. Testosterone concentrations in humans and mice were assessed by RIA.

### Statistical Analysis

Samples were analyzed by one-way ANOVA, with Bonferroni correction if there were more than two groups, and two-tailed Student's *t* tests were used for pairwise comparisons (WT versus KO). Statistical analyses were carried out with Prism software (GraphPad, La Jolla, CA). Differences were considered significant if *p*<0.05.

## Supporting Information

Figure S1 Sanger sequencing analysis of exon 24 of *DMXL2* in the family. Extracts of Sanger sequencing chromatograms for exon 24 of *DMXL2* for one affected patient (#123), one heterozygous parent (#121), and one WT sister (#127). Primer sequences are available upon request. The in-frame deleted region in exon 24 is underlined in the chromatogram of patient #127. *DMXL2* was identified as an interesting candidate gene by the next-generation sequencing of two candidate regions delineated by genome mapping. All exons of these two regions were captured from the proband DNA and his mother DNA and sequenced with the 454 sequencing system. We found 3,295 single-nucleotide polymorphisms in the two DNA samples, 2,015 of which were common to the proband and his mother. Among these 2,015 SNPs, 1,612 had previously been reported in HapMap and dbSNP. Of the 403 unknown SNPs in these two databases, 91 were present in mRNAs, but only 20 were present in coding sequences. Six of these variants were considered to be of interest. These variants were located in *ZNF770*, *MTMR15*, *TMCO5A*, and *DMXL2* (Table S7). *ZNF770*, *MTMR15*, and *TMCO5A* were rejected, as the variants concerned were reported in the 1000 Genomes database (browser.1000genomes.org) and/or the substituent amino acids were present in the proteins of other species. Applying the criteria described above, none of the DNA variants found on chromosome 13 were considered valid candidates. This left only one potentially interesting variant in *DMXL2* on chromosome 15. This DNA variant was an in-frame deletion of 15 nucleotides (c.5824_5838del) in exon 24, leading to a deletion of five amino-acid residues from the protein (p.1942_1946del). A similar but not identical deletion (c.5827-5842del16) has recently been reported in the exome variant server (http://evs.gs.washington.edu), with a frequency of 3/10,000 but without the identification of a homozygous carrier. The sequence shown is the coding sequence. DMXL2 is encoded by the minus strand. M, mutated allele; wt, WT allele.
(EPS)

Figure S2 Analysis of Rbcn-3α expression in the hypothalamus and cerebellum. IHC was performed on floating sections as described in “Materials and Methods.” (A) Rbcn-3α staining was observed in the granular layer (GL) as well as molecular layer (ML) in the cerebellum. Note that purkinje cells do not express Rbcn-3α (white arrow heads). (B) Rbcn-3α immunostaining was observed in the SCN as well as along the third ventricle (V3) in the periventricular nucleus (Pe). (C and D) A positive staining was also observed in the SFO and the subcomissural organ (CMO). Black arrow heads indicate positive staining.
(TIF)

Figure S3 The EUCOMM *Dmxl2* gene trap allele with conditional potential and the *Dmxl2* allele after the action of Cre recombinase. (A) The *Dmxl2* gene trap allele (tm1a allele) contains an IRES:*LacZ* trapping cassette with an acceptor splice site (En2 SA) and a floxed *neo^R^* cassette. *LoxP* sites flank the critical exon 7 and the *neo^R^* cassette. FRT sites flank the IRES:*LacZ* and *neo^R^* cassettes (adapted from www.knockoutmouse.org/about/eucomm). The IRES:*LacZ* trapping cassette and the floxed *neo^R^* cassette have been deleted by the FLIP recombinase to generate *Dmxl2^lox/lox^* mice. The critical exon 7 was then deleted by the Nestin cre-recombinase to generate both *Dmxl2^lox/wt^* (WT) and *nes-Cre;Dmxl2^–/wt^*. Dashed lines indicate possible RNA splice isoforms. Single-headed orange or blue arrows represent the positions of the PCR primers used to genotype mice. (B) Relative expression of *Dmxl2* in the hypothalamus of *nes-Cre;Dmxl2^–/wt^* mice; comparison with results for WT littermates. Total RNA was extracted from the dissected hypothalamus of *nes-Cre;Dmxl2^–/wt^* and *Dmxl2^lox/wt^* mice and reverse-transcribed with random primers. The *Dmxl2* cDNA was then quantified by qPCR, as described in “Materials and Methods.” *** *p*<0. 001. White bar, *Dmxl2^lox/wt^* mice; black bar, *nes-Cre;Dmxl2^–/wt^* mice. Numerical data used to generate graph S3B may be found in Table S5.
(EPS)

Figure S4 Analysis of the age at VO and the time between VO and the age at the first estrus in *Dmxl2^tm1a/wt^* mice. (A) A tendency toward an older age at VO. (B) A higher time between VO and the age at the first estrus was observed in *Dmxl2^tm1a/wt^* mice (gray bars) as compared to WT littermates (white bars). Numerical data used to generate these two graphs may be found in Table S5.
(EPS)

Figure S5 Growth curve of *nes-Cre;Dmxl2^–/wt^* male mice as compared to their WT littermates *Dmxl2^lox/wt^* mice. Numerical data used to generate this graph may be found in Table S6.
(EPS)

Figure S6 Quantification of corticotropin-releasing hormone (CRH) and thyrotropin-releasing hormone (TRH) mRNA levels in the hypothalamus of *nes-Cre;Dmxl2^–/wt^* mice. Hypothalami of *nes-Cre;Dmxl2^–/wt^* and *Dmxl2^lox/wt^* mice were dissected, and total RNA was extracted as described in “Materials and Methods.” Levels of CRH and TRH mRNA were assessed by quantitative RT-qPCR. Primer sequences are available on request. CRH, corticotropin-releasing hormone; TRH, thyrotropin-releasing hormone. White bars, *Dmxl2^lox/wt^* mice; black bars, *nes-Cre;Dmxl2^–/wt^* mice. Numerical data used to generate these two graphs may be found in Table S5.
(EPS)

Figure S7 Investigation of gait and exploratory behavior in nes-Cre;Dmxl2^–/wt^ mice. The Neogait Openfield system was used for automatic measurement of the gait and exploratory behavior of Dmxl2^lox/wt^ and nes-Cre;Dmxl2^–/wt^ mice. Gait was assessed by measuring two variables: distance from the starting point to the end point and the distance covered (number of blocks occupied). No significant differences in gait were found between any of the genotypes tested. However, there was a significant difference in the number of blocks occupied, indicating higher levels of exploratory behavior in nes-Cre;Dmxl2^–/wt^ mice than in their WT littermates. This difference disappeared in the second trial, suggesting a possible effect of fatigue, as the two trials were separated by an interval of only about 20 min. White and black bars represent the means of blocks occupied in trials 1 and 2 by nes-Cre;Dmxl2^–/wt^ mice or their WT litter mates, respectively (see numerical data in Table S5).
(EPS)

Table S1 Body weight in *nes-cre;Dmxl2* ^tm1a/wt^ mice. The body weights of male *Dmxl2* ^tm1a/wt^ and *Dmxl2* ^wt/wt^ mice were not significantly different. PND, postnatal day.
(DOC)

Table S2 Fertility analysis in nes-Cre;*Dmxl2* ^tm1a/wt^ mice. Total number of litters, pups, sex of pups, genotype of pups, and mean litter size for all mice analyzed for this study over a 6-mo period.
(DOC)

Table S3 Fertility tests in nesCre;*Dmxl2* ^–/wt^ mice.
(DOC)

Table S4 Antibodies used for IHC.
(DOC)

Table S5 Numerical data used to generate graphs. These data were those used to generate graphs and to perform statistical analyses. In Figures 4B, 4C, 4D, 4E, S4A, and S4B, units are postnatal days. In Figure 4F, units are the time spent in each estradiol concentration (percentage) in one ovarian cycle. In Figure 5A and 5B, units are the means of the weight of both gonads (milligrams). In Figure 5C, units are the mean number of follicles observed in ovaries. In Figure 5D and 5I, units are estradiol concentrations in pg/ml. In Figure 5E, units are testosterone concentrations in ng/ml. In Figure 5F–H, units are LH concentrations in ng/ml. In Figure 1D, units are *DMXL2* mRNA levels relative to *RNA 18S* in blood lymphocytes of patients and controls (100×). In Figures 6C, S3B, S6A, and S6B, units are mRNA relative values to the mean measured in *Dmxl2^lox/wt^* mice. In Figure 7B, units are *Dmxl2* mRNA levels relative to *RNA18S* levels (100×). In Figure 7C, units are the insulin concentrations in ng/ml. In Figure 6D, 6F, and 6F inset, units are the number of GnRH-IR neurons. In qPCR analysis, Dup1 and Dup2 indicate duplicate 1 and duplicate 2, respectively.
(XLSX)

Table S6 Numerical data used to draw graphs of the postnatal growth in both sexes and the AGDs in males. In Figures 4A and S5, data are the weights of mice in grams. In Figure 4G, data are the distance in millimeters.
(XLSX)

Table S7 List of six selected DNA variants in the two candidates regions. The selection criteria were those defined in the legend of Figure S1. The genome reference is hg18.
(XLSX)

## Abbreviations

ACTH: adrenocorticotropic hormone
AGD: anogenital distance
ARC: arcuate nucleus
CHH: congenital hypogonatropic hypogonadism
CNS: central nervous system
CRH: corticotropin-releasing hormone
FSH: follicle-stimulating hormone
GABA: γ-aminobutyric acid
GH: growth hormone
GnRH: gonadotropin-releasing hormone
HPG: hypothalamp–pituitary–gonadotropic
KP: kisspeptin
KS: Kallmann syndrome
LH: luteinizing hormone
ME: median eminence
NKB: Neurokinin B
OP: olfactory placode
OVLT: organum vasculosum of the lamina terminalis
Pe: periventricular nucleus
PMSG: pregnant mare's serum gonadotropin
POA: preoptic area
Rbcn-3α: Rabconnectin-3α
SNC: suprachiasmatic nucleus
SCO: subcommissural organ
SFO: subfornical organ
TRH: thyreotropin-releasing hormone
TSH: thyreo-stimulating hormone
VO: vaginal opening

## References

[1] Sisk CL, Foster DL (2004) The neural basis of puberty and adolescence. Nat Neurosci 7: 1040–1047. 15452575 10.1038/nn1326

[2] Plant TM, Witchel SF (2006) Puberty in nonhuman primates and humans. In: Neill JD, editor. Knobil and Neil's physiology of reproduction. St Louis, MO: Elsevier. pp. 2177–2230.

[3] Iremonger KJ, Constantin S, Liu X, Herbison AE (2010) Glutamate regulation of GnRH neuron excitability. Brain Res 1364: 35–43. 20807514 10.1016/j.brainres.2010.08.071

[4] Ojeda SR, Lomniczi A, Sandau U, Matagne V (2010) New concepts on the control of the onset of puberty. Endocr Dev 17: 44–51. 19955755 10.1159/000262527

[5] Pinilla L, Aguilar E, Dieguez C, Millar RP, Tena-Sempere M (2012) Kisspeptins and reproduction: physiological roles and regulatory mechanisms. Physiol Rev 92: 1235–1316. 22811428 10.1152/physrev.00037.2010

[6] Goodman RL, Lehman MN (2012) Kisspeptin neurons from mice to men: similarities and differences. Endocrinology 153: 5105–5118. 22989628 10.1210/en.2012-1550 PMC3473207

[7] Lomniczi A, Loche A, Castellano JM, Ronnekleiv OK, Bosch M, et al (2013) Epigenetic control of female puberty. Nat Neurosci 16: 281–289. 23354331 10.1038/nn.3319 PMC3581714

[8] de Roux N, Young J, Misrahi M, Genet R, Chanson P, et al (1997) A family with hypogonadotropic hypogonadism and mutations in the gonadotropin-releasing hormone receptor. N Engl J Med 337: 1597–1602. 9371856 10.1056/NEJM199711273372205

[9] de Roux N, Genin E, Carel JC, Matsuda F, Chaussain JL, et al (2003) Hypogonadotropic hypogonadism due to loss of function of the KiSS1-derived peptide receptor GPR54. Proc Natl Acad Sci U S A 100: 10972–10976. 12944565 10.1073/pnas.1834399100 PMC196911

[10] Seminara SB, Messager S, Chatzidaki EE, Thresher RR, Acierno JS Jr, et al (2003) The GPR54 gene as a regulator of puberty. N Engl J Med 349: 1614–1627. 14573733 10.1056/NEJMoa035322

[11] Topaloglu AK, Tello JA, Kotan LD, Ozbek MN, Yilmaz MB, et al (2012) Inactivating KISS1 mutation and hypogonadotropic hypogonadism. N Engl J Med 366: 629–635. 22335740 10.1056/NEJMoa1111184

[12] Topaloglu AK, Reimann F, Guclu M, Yalin AS, Kotan LD, et al (2009) TAC3 and TACR3 mutations in familial hypogonadotropic hypogonadism reveal a key role for Neurokinin B in the central control of reproduction. Nat Genet 41: 354–358. 19079066 10.1038/ng.306 PMC4312696

[13] Navarro VM (2013) Interactions between kisspeptins and neurokinin B. Adv Exp Med Biol 784: 325–347. 23550013 10.1007/978-1-4614-6199-9_15 PMC3858905

[14] Dode C, Hardelin JP (2010) Clinical genetics of Kallmann syndrome. Ann Endocrinol (Paris) 71: 149–157. 20362962 10.1016/j.ando.2010.02.005

[15] Pitteloud N, Durrani S, Raivio T, Sykiotis GP (2010) Complex genetics in idiopathic hypogonadotropic hypogonadism. Front Horm Res 39: 142–153. 20389092 10.1159/000312700

[16] Wray S (2010) From nose to brain: development of gonadotrophin-releasing hormone-1 neurones. J Neuroendocrinol 22: 743–753. 20646175 10.1111/j.1365-2826.2010.02034.x PMC2919238

[17] Alazami AM, Al-Saif A, Al-Semari A, Bohlega S, Zlitni S, et al (2008) Mutations in C2orf37, encoding a nucleolar protein, cause hypogonadism, alopecia, diabetes mellitus, mental retardation, and extrapyramidal syndrome. Am J Hum Genet 83: 684–691. 19026396 10.1016/j.ajhg.2008.10.018 PMC2668059

[18] Aligianis IA, Johnson CA, Gissen P, Chen D, Hampshire D, et al (2005) Mutations of the catalytic subunit of RAB3GAP cause Warburg Micro syndrome. Nat Genet 37: 221–223. 15696165 10.1038/ng1517

[19] Aligianis IA, Morgan NV, Mione M, Johnson CA, Rosser E, et al (2006) Mutation in Rab3 GTPase-activating protein (RAB3GAP) noncatalytic subunit in a kindred with Martsolf syndrome. Am J Hum Genet 78: 702–707. 16532399 10.1086/502681 PMC1424696

[20] Bernard G, Chouery E, Putorti ML, Tetreault M, Takanohashi A, et al (2011) Mutations of POLR3A encoding a catalytic subunit of RNA polymerase Pol III cause a recessive hypomyelinating leukodystrophy. Am J Hum Genet 89: 415–423. 21855841 10.1016/j.ajhg.2011.07.014 PMC3169829

[21] Tetreault M, Choquet K, Orcesi S, Tonduti D, Balottin U, et al (2011) Recessive mutations in POLR3B, encoding the second largest subunit of Pol III, cause a rare hypomyelinating leukodystrophy. Am J Hum Genet 89: 652–655. 22036172 10.1016/j.ajhg.2011.10.006 PMC3213403

[22] Houseley J, Tollervey D (2009) The many pathways of RNA degradation. Cell 136: 763–776. 19239894 10.1016/j.cell.2009.01.019

[23] Kawabe H, Sakisaka T, Yasumi M, Shingai T, Izumi G, et al (2003) A novel rabconnectin-3-binding protein that directly binds a GDP/GTP exchange protein for Rab3A small G protein implicated in Ca(2+)-dependent exocytosis of neurotransmitter. Genes Cells 8: 537–546. 12786944 10.1046/j.1365-2443.2003.00655.x

[24] Nagano F, Kawabe H, Nakanishi H, Shinohara M, Deguchi-Tawarada M, et al (2002) Rabconnectin-3, a novel protein that binds both GDP/GTP exchange protein and GTPase-activating protein for Rab3 small G protein family. J Biol Chem 277: 9629–9632. 11809763 10.1074/jbc.C100730200

[25] Sethi N, Yan Y, Quek D, Schupbach T, Kang Y (2010) Rabconnectin-3 is a functional regulator of mammalian Notch signaling. J Biol Chem 285: 34757–34764. 20810660 10.1074/jbc.M110.158634 PMC2966091

[26] Skarnes WC, Rosen B, West AP, Koutsourakis M, Bushell W, et al (2011) A conditional knockout resource for the genome-wide study of mouse gene function. Nature 474: 337–342. 21677750 10.1038/nature10163 PMC3572410

[27] Skarnes WC (2005) Two ways to trap a gene in mice. Proc Natl Acad Sci U S A 102: 13001–13002. 16150712 10.1073/pnas.0506279102 PMC1201614

[28] Guan C, Ye C, Yang X, Gao J (2010) A review of current large-scale mouse knockout efforts. Genesis 48: 73–85. 20095055 10.1002/dvg.20594

[29] Dubois NC, Hofmann D, Kaloulis K, Bishop JM, Trumpp A (2006) Nestin-Cre transgenic mouse line Nes-Cre1 mediates highly efficient Cre/loxP mediated recombination in the nervous system, kidney, and somite-derived tissues. Genesis 44: 355–360. 16847871 10.1002/dvg.20226

[30] Dehorter N, Michel FJ, Marissal T, Rotrou Y, Matrot B, et al (2011) Onset of Pup locomotion coincides with loss of NR2C/D-mediated cortico-striatal EPSCs and dampening of striatal network immature activity. Front Cell Neurosci 5: 24. 22125512 10.3389/fncel.2011.00024 PMC3221398

[31] Hutagalung AH, Novick PJ (2011) Role of Rab GTPases in membrane traffic and cell physiology. Physiol Rev 91: 119–149. 21248164 10.1152/physrev.00059.2009 PMC3710122

[32] Burgoyne RD, Morgan A (2003) Secretory granule exocytosis. Physiol Rev 83: 581–632. 12663867 10.1152/physrev.00031.2002

[33] Jacobsson G, Bean AJ, Scheller RH, Juntti-Berggren L, Deeney JT, et al (1994) Identification of synaptic proteins and their isoform mRNAs in compartments of pancreatic endocrine cells. Proc Natl Acad Sci U S A 91: 12487–12491. 7809063 10.1073/pnas.91.26.12487 PMC45463

[34] Handley MT, Aligianis IA (2012) RAB3GAP1, RAB3GAP2 and RAB18: disease genes in Micro and Martsolf syndromes. Biochem Soc Trans 40: 1394–1397. 23176487 10.1042/BST20120169

[35] Wierman ME, Kiseljak-Vassiliades K, Tobet S (2011) Gonadotropin-releasing hormone (GnRH) neuron migration: initiation, maintenance and cessation as critical steps to ensure normal reproductive function. Front Neuroendocrinol 32: 43–52. 20650288 10.1016/j.yfrne.2010.07.005 PMC3008544

[36] Tuttle AM, Hoffman TL, Schilling TF (2014) Rabconnectin-3a regulates vesicle endocytosis and canonical Wnt signaling in zebrafish neural crest migration. PLoS Biol 12: e1001852. 24802872 10.1371/journal.pbio.1001852 PMC4011682

[37] Carel JC, Boitard C, Bougneres PF (1993) Decreased insulin response to glucose in islet cell antibody-negative siblings of type 1 diabetic children. J Clin Invest 92: 509–513. 8326015 10.1172/JCI116595 PMC293639

[38] Huyghe JR, Jackson AU, Fogarty MP, Buchkovich ML, Stancakova A, et al (2013) Exome array analysis identifies new loci and low-frequency variants influencing insulin processing and secretion. Nat Genet 45: 197–201. 23263489 10.1038/ng.2507 PMC3727235

[39] Dupuis J, Langenberg C, Prokopenko I, Saxena R, Soranzo N, et al (2010) New genetic loci implicated in fasting glucose homeostasis and their impact on type 2 diabetes risk. Nat Genet 42: 105–116. 20081858 10.1038/ng.520 PMC3018764

[40] Izumi T, Gomi H, Kasai K, Mizutani S, Torii S (2003) The roles of Rab27 and its effectors in the regulated secretory pathways. Cell Struct Funct 28: 465–474. 14745138 10.1247/csf.28.465

[41] Geppert M, Goda Y, Stevens CF, Sudhof TC (1997) The small GTP-binding protein Rab3A regulates a late step in synaptic vesicle fusion. Nature 387: 810–814. 9194562 10.1038/42954

[42] Sakane A, Manabe S, Ishizaki H, Tanaka-Okamoto M, Kiyokage E, et al (2006) Rab3 GTPase-activating protein regulates synaptic transmission and plasticity through the inactivation of Rab3. Proc Natl Acad Sci U S A 103: 10029–10034. 16782817 10.1073/pnas.0600304103 PMC1502500

[43] Geppert M, Bolshakov VY, Siegelbaum SA, Takei K, De Camilli P, et al (1994) The role of Rab3A in neurotransmitter release. Nature 369: 493–497. 7911226 10.1038/369493a0

[44] Figueiredo AC, Wasmeier C, Tarafder AK, Ramalho JS, Baron RA, et al (2008) Rab3GEP is the non-redundant guanine nucleotide exchange factor for Rab27a in melanocytes. J Biol Chem 283: 23209–23216. 18559336 10.1074/jbc.M804134200 PMC2516999

[45] Yamaguchi K, Tanaka M, Mizoguchi A, Hirata Y, Ishizaki H, et al (2002) A GDP/GTP exchange protein for the Rab3 small G protein family up-regulates a postdocking step of synaptic exocytosis in central synapses. Proc Natl Acad Sci U S A 99: 14536–14541. 12388783 10.1073/pnas.212511399 PMC137918

[46] Yan Y, Denef N, Schupbach T (2009) The vacuolar proton pump, V-ATPase, is required for notch signaling and endosomal trafficking in Drosophila. Dev Cell 17: 387–402. 19758563 10.1016/j.devcel.2009.07.001 PMC2758249

[47] Einhorn Z, Trapani JG, Liu Q, Nicolson T (2012) Rabconnectin3alpha promotes stable activity of the H+ pump on synaptic vesicles in hair cells. J Neurosci 32: 11144–11156. 22875945 10.1523/JNEUROSCI.1705-12.2012 PMC3428958

[48] Ruschendorf F, Nurnberg P (2005) ALOHOMORA: a tool for linkage analysis using 10K SNP array data. Bioinformatics 21: 2123–2125. 15647291 10.1093/bioinformatics/bti264

[49] Gudbjartsson DF, Thorvaldsson T, Kong A, Gunnarsson G, Ingolfsdottir A (2005) Allegro version 2. Nat Genet 37: 1015–1016. 16195711 10.1038/ng1005-1015

[50] Khalfallah O, Faucon-Biguet N, Nardelli J, Meloni R, Mallet J (2008) Expression of the transcription factor Zfp191 during embryonic development in the mouse. Gene Expr Patterns 8: 148–154. 18096443 10.1016/j.gep.2007.11.002

[51] Villanueva C, Jacquier S, de Roux N (2012) DLK1 is a somato-dendritic protein expressed in hypothalamic arginine-vasopressin and oxytocin neurons. PLoS ONE 7: e36134. 22563444 10.1371/journal.pone.0036134 PMC3338567

[52] Wray S, Gahwiler BH, Gainer H (1988) Slice cultures of LHRH neurons in the presence and absence of brainstem and pituitary. Peptides 9: 1151–1175. 3072535 10.1016/0196-9781(88)90103-9

[53] Chung WC, Matthews TA, Tata BK, Tsai PS (2010) Compound deficiencies in multiple fibroblast growth factor signalling components differentially impact the murine gonadotrophin-releasing hormone system. J Neuroendocrinol 22: 944–950. 20553372 10.1111/j.1365-2826.2010.02024.x PMC3102046

[54] Dumoulin A, Rostaing P, Bedet C, Levi S, Isambert MF, et al (1999) Presence of the vesicular inhibitory amino acid transporter in GABAergic and glycinergic synaptic terminal boutons. J Cell Sci 112 (Pt 6) 811–823. 10036231 10.1242/jcs.112.6.811

[55] Merglen A, Theander S, Rubi B, Chaffard G, Wollheim CB, et al (2004) Glucose sensitivity and metabolism-secretion coupling studied during two-year continuous culture in INS-1E insulinoma cells. Endocrinology 145: 667–678. 14592952 10.1210/en.2003-1099

[56] Tosca L, Froment P, Rame C, McNeilly JR, McNeilly AS, et al (2011) Metformin decreases GnRH- and activin-induced gonadotropin secretion in rat pituitary cells: potential involvement of adenosine 5′ monophosphate-activated protein kinase (PRKA). Biol Reprod 84: 351–362. 20980683 10.1095/biolreprod.110.087023

[57] Seol JH, Shevchenko A, Shevchenko A, Deshaies RJ (2001) Skp1 forms multiple protein complexes, including RAVE, a regulator of V-ATPase assembly. Nat Cell Biol 3: 384–391. 11283612 10.1038/35070067

