# Supplementary figures and images for: Haploinsufficiency of Dmxl2, Encoding a Synaptic Protein, Causes Infertility Associated with a Loss of GnRH Neurons in Mouse
Source: PLoS Biol. 2014 Sep 23;12(9):e1001952. doi: 10.1371/journal.pbio.1001952 (PMC4172557; doi:10.1371/journal.pbio.1001952)

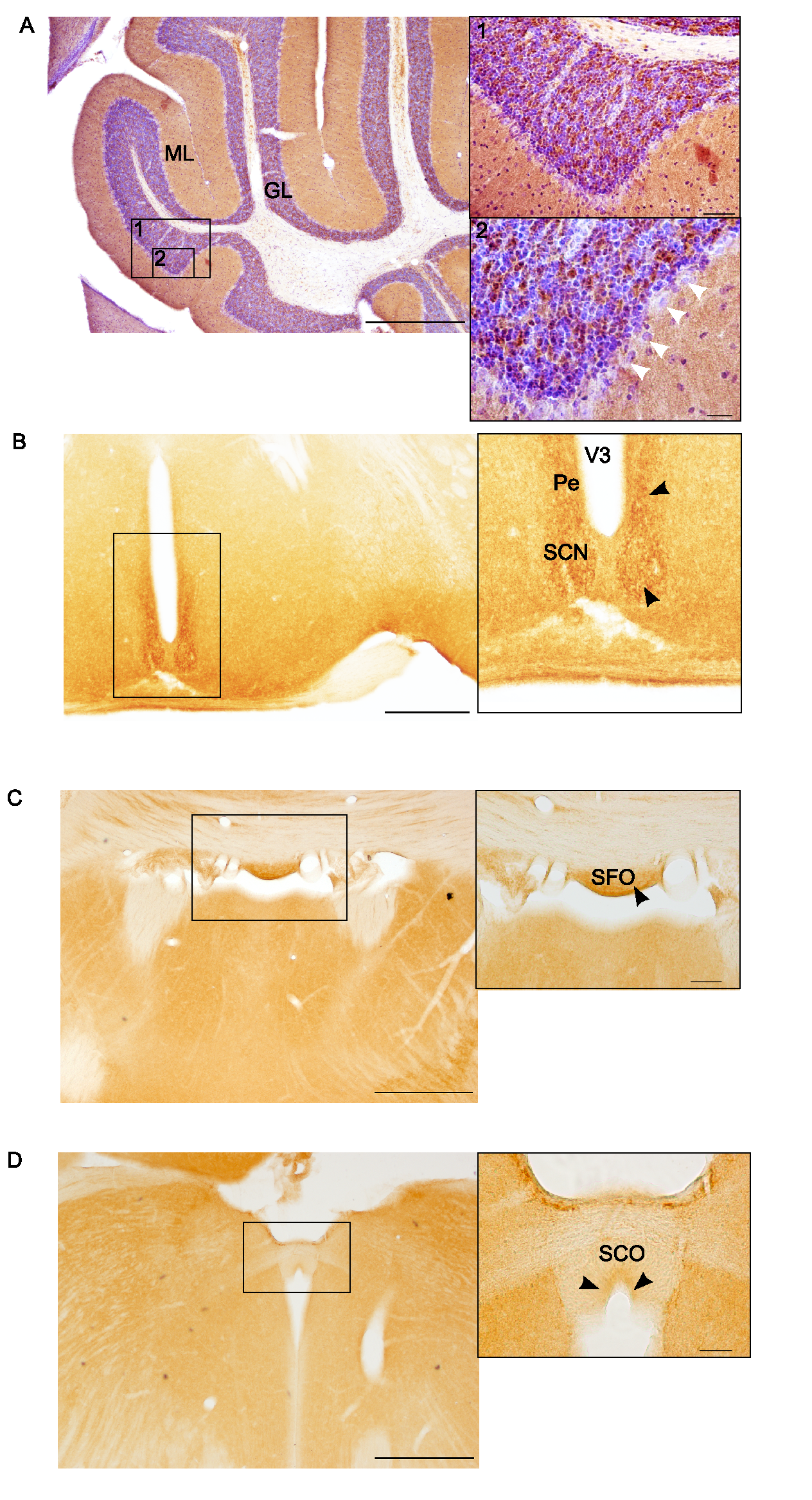

Supplement: Figure S2 — Analysis of Rbcn-3α expression in the hypothalamus and cerebellum. IHC was performed on floating sections as described in “Materials and Methods.” (A) Rbcn-3α staining was observed in the granular layer (GL) as well as molecular layer (ML) in the cerebellum. Note that purkinje cells do not express Rbcn-3α (white arrow heads). (B) Rbcn-3α immunostaining was observed in the SCN as well as along the third ventricle (V3) in the periventricular nucleus (Pe). (C and D) A positive staining was also observed in the SFO and the subcomissural organ (CMO). Black arrow heads indicate positive staining. (TIF) [file pbio.1001952.s002.tif]
