# Supplementary material for: Haploinsufficiency of Dmxl2, Encoding a Synaptic Protein, Causes Infertility Associated with a Loss of GnRH Neurons in Mouse
Source: PLoS Biol. 2014 Sep 23;12(9):e1001952. doi: 10.1371/journal.pbio.1001952 (PMC4172557; doi:10.1371/journal.pbio.1001952)
Supplement: Table S1 — Body weight in nes-cre;Dmxl2 tm1a/wt mice. The body weights of male Dmxl2 tm1a/wt and Dmxl2 wt/wt mice were not significantly different. PND, postnatal day. (DOC) [file pbio.1001952.s008.doc]

**Table S1.**

| Age (PND) | *Dmxl2^wt/wt^* | *Dmxl2*^tm1a/wt^ |
| --- | --- | --- |
| 30 | 15.07 g ± 1.15 | 17.45 g ± 1.06 |
| 35 | 19.26 g ± 1.11 | 19.73 g ± 1.38 |
| 40 | 20.85 g ± 1.07 | 21.48 g ± 1.44 |
| 45 | 22.29 g ± 1.07 | 22.71 g ± 1.47 |
