# Supplementary material for: Haploinsufficiency of Dmxl2, Encoding a Synaptic Protein, Causes Infertility Associated with a Loss of GnRH Neurons in Mouse
Source: PLoS Biol. 2014 Sep 23;12(9):e1001952. doi: 10.1371/journal.pbio.1001952 (PMC4172557; doi:10.1371/journal.pbio.1001952)
Supplement: Table S2 — Fertility analysis in nes-Cre;Dmxl2 tm1a/wt mice. Total number of litters, pups, sex of pups, genotype of pups, and mean litter size for all mice analyzed for this study over a 6-mo period. (DOC) [file pbio.1001952.s009.doc]

**Table S2**

|  | *Dmxl2*^tm1a/wt^  x  *Dmxl2*^tm1a/wt^ |
| --- | --- |
| Total # of litters/6 months | 5 |
| Total # of pups/6 months | 22 |
| Sex of pups | 10 females  12 males |
| Genotype of pups | 9 *Dmxl2^wt/wt^*  13 *Dmxl2*^tm1a/wt^  0 *Dmxl2^tm1a/tm1a^* |
| Mean litter size/6 months | 4.2 pups/litter |

|  |  |  |  |
| --- | --- | --- | --- |
