# Supplementary material for: Haploinsufficiency of Dmxl2, Encoding a Synaptic Protein, Causes Infertility Associated with a Loss of GnRH Neurons in Mouse
Source: PLoS Biol. 2014 Sep 23;12(9):e1001952. doi: 10.1371/journal.pbio.1001952 (PMC4172557; doi:10.1371/journal.pbio.1001952)
Supplement: Table S3 — Fertility tests in nesCre;Dmxl2 –/wt mice. (DOC) [file pbio.1001952.s010.doc]

**Table S3**

|  | *Nes-Cre ;Dmxl2 ^lox/wt^ (f)*  *X*  *Nes-Cre ;Dmxl2 ^lox/wt^(m)* | *Nes-Cre ;Dmxl2 ^lox/wt^ (f)*  *X*  *Nes-Cre ;Dmxl2 ^-/wt^ (m)* | *Nes-Cre ;Dmxl2 ^lox/wt^ (m)*  *X*  *Nes-Cre ;Dmxl2 ^-/wt^ (f)* |
| --- | --- | --- | --- |
| Pups | 33.1 ± 2.6 | 17.4 ± 2.7 | 9.4 ± 1.4 |
| Litters | 4 ± 0.3 | 2.6 ± 0.5 | 2.0 ± 0.3 |
| Pups/litter | 8.3 ± 0.4 | 6.8 ± 0.4 | 4.5 ± 0.2 |
| Time to 1^st^ litter | 21.5 ± 0.4 | 27.1 ± 3.0 | 33.9 ± 2.9 |
