# Supplementary material for: Haploinsufficiency of Dmxl2, Encoding a Synaptic Protein, Causes Infertility Associated with a Loss of GnRH Neurons in Mouse
Source: PLoS Biol. 2014 Sep 23;12(9):e1001952. doi: 10.1371/journal.pbio.1001952 (PMC4172557; doi:10.1371/journal.pbio.1001952)
Supplement: Table S4 — Antibodies used for IHC. (DOC) [file pbio.1001952.s011.doc]

**Table S4.**

| Antibody | Company | Dilution |
| --- | --- | --- |
| Guinea pig antiserum against rat GH | NIDK | 1:2000 |
| Guinea pig antiserum against rat βLH | NIDK | 1:2000 |
| Guinea pig antiserum against rat βFSH | NIDK | 1:2000 |
| Guinea pig antiserum against rat βTSH | NIDK | 1:2000 |
| Guinea pig antiserum against rat ACTH | NIDK | 1:6000 |
| Mouse antiserum against mouse GnRH | Gift from Susan Wray^1^ | 1:200 |
| Rabbit polyclonal anti-Dmxl2 antibody | Sigma | 1:400 |
| Rabbit polyclonal anti-MADD antibody (H-300) | Santa Cruz | 1:200 |
| Goat polyclonal anti-Rab3GAP p130 antibody (T-16) | Santa Cruz | 1:100 |
| Guinea pig polyclonal anti-insulin antibody | Dako | 1:400 |
|  |  |  |

1 Wray S, Gahwiler BH, Gainer H (1988) Slice cultures of LHRH neurons in the presence and absence of brainstem and pituitary. Peptides 9: 1151-1175.
